# Birthing balls and peanut balls for labor pain, delivery duration, and mode of delivery: a meta-analysis of randomized controlled trials

**DOI:** 10.7717/peerj.21062

**Published:** 2026-04-02

**Authors:** Qiuhong Luo, Xiao Feng, Meng Zhang, Yan Wu, Linyan Wu, Mingying Tan

**Affiliations:** 1 Outpatient Department, West China Tianfu Hospital, Sichuan University, Chengdu, China; 2 Outpatient Department, West China Hospital, Sichuan University, Chengdu, China

**Keywords:** Birthing ball, Peanut ball, Labor, Delivery, Natural childbirth

## Abstract

**Background:**

To determine the effects of birthing balls and peanut balls on labor pain, duration of labor, and mode of delivery.

**Methods:**

In this systematic review and meta-analysis, Cochrane Library, EMBASE, MEDLINE (*via* PubMed), Scopus, and Web of Science were searched from their inception to December 25, 2024, with an updated search performed on November 10, 2025. Included studies were randomized controlled trials written in English that investigated the efficacy of birthing balls or peanut balls compared to no birthing balls or peanut balls in pregnant women with a singleton and cephalic fetus. Conference abstract, editorial, review, dissertation, thesis, and non-randomized controlled trials were excluded. Primary outcomes were labor pain and duration of labor, while the secondary outcome was mode of delivery.

**Results:**

A total of 23 studies involving 3,192 participants were included. Relative to no birthing balls, birthing balls significantly alleviated labor pain (MD: −1.81; 95% CI [−2.38 to −1.23]), shortened the duration of the first (SMD: −0.86; −1.26 to −0.45) and second (MD: −17.00 min; −26.54 to −7.46) stages of labor, and reduced the incidence of cesarean delivery (RR: 0.51; 0.37 to 0.71). Peanut balls significantly shortened the duration of the first (SMD: −0.85; −1.25 to −0.46) and second (MD: −10.90 min; −21.11 to −0.70) stages of labor, compared to no peanut balls. No other significant results were observed. Some concerns were raised and the overall quality ranged from moderate to low.

**Conclusions:**

Based on low- to moderate-quality evidence, birthing balls appear to be a potentially effective tool and may be considered by nurses and midwives to help alleviate labor pain and shorten the duration of the first and second stages of labor, thereby potentially reducing the likelihood of cesarean delivery. Low-quality evidence suggests that peanut balls could help shorten the duration of the first and second stages of labor. More well-designed studies are needed to verify the true and comparative effects of birthing and peanut balls.

## Introduction

Persistent and intense pain during labor and delivery can negatively impact both the laboring women and fetus, and may even alter the course of childbirth (Delgado et al., 2024; De Sena Fraga et al., 2024). Extended labor and failure to progress usually increase the likelihood of a cesarean section, (Grenvik et al., 2019) and are associated with maternal (*e.g.*, chorioamnionitis, postpartum hemorrhage) and neonatal complications (*e.g.*, neonatal intensive care unit admission) (Ahmadpour et al., 2021). Therefore, reducing labor pain and shortening the duration of labor are of significance in clinical scenarios (Aquino et al., 2024). Lack of maternal position changes during labor reduces the likelihood of an ideal birth, elevating the likelihood of prolonged labor, labor arrest, and cesarean delivery (Aquino et al., 2024; Kamath et al., 2024). The World Health Organization recommends non-pharmacological modalities, such as freedom of position, non-supine postures, and movements during labor and delivery, to improve perinatal outcomes (Smith et al., 2018b; WH Organization, 2018). Consistent with this recommendation, encouraging laboring women to perform upright positioning and increase maternal mobility has been shown to confer multiple benefits during labor, as evidenced by previous studies (Hemmerich, Bandrowska & Dumas, 2019; Morales-Alvarado & Paredes-Pérez, 2024; Reitter et al., 2014; Walker et al., 2018).

The birthing ball is a spherical plastic exercise ball (also known as a Swiss ball), and the peanut ball is an elongated, inflatable birthing ball with a narrowed midsection. This unique structural design of the peanut ball enables it to better conform to the maternal pelvic contour, providing stable support during labor while allowing greater freedom of pelvic rotation and hip abduction. Such positioning advantages are hypothesized to facilitate fetal descent and engagement, relieve pelvic floor muscle tension, and optimize uterine contraction coordination, all of which align with the core goals of an ideal birth (Aquino et al., 2024). Both birthing balls and peanut balls are non-pharmacological modalities believed to mimic upright positioning and promote maternal mobility during labor, thereby exerting therapeutic effects similar to those of the aforementioned interventions (Hemmerich, Bandrowska & Dumas, 2019; Morales-Alvarado & Paredes-Pérez, 2024; Reitter et al., 2014; Walker et al., 2018). During labor, women sit on these balls and perform various movements (*e.g.*, forward–backward rocking, pelvic rotation) as well as adopt different positions (De Sena Fraga et al., 2024; Erkal Aksoy, Dereli Yilmaz & Çelimli, 2024). However, despite the consistency of recommendations for the use of birthing balls and peanut balls during labor, there are inconsistent findings in previous trials (Gau et al., 2011; Taavoni et al., 2011; Mercier & Kwan, 2018; Roth et al., 2016) and uncertainty in previous reviews (Delgado et al., 2022; Grenvik et al., 2022). Specifically, while systematic reviews of evidence available support birthing balls to decrease labor pain and peanut balls to decrease the duration of labor (Delgado et al., 2022; Grenvik et al., 2022), it is limited by the small sample size and has an uncertain efficacy for other outcomes. Considering that it is a rapidly evolving area, and a number of trials in birthing balls and peanut balls for labor and delivery have been published (Kamath et al., 2024; Alan Dikmen, Gönenç & Ataş, 2024; Mylod et al., 2024) since the last systematic review on this topic in 2020 (Delgado et al., 2022), an up-to-date meta-analysis is warranted.

Therefore, agreed with the Population, Intervention, Comparison, and Outcomes (PICO) elements, the specific research question for this meta-analysis was: In laboring women with a singleton and cephalic fetus, how effective are birthing balls or peanut balls compared to standard care without these devices in terms of labor pain, labor duration, and mode of delivery? We hypothesize they may reduce pain, shorten duration of labor, and favor vaginal delivery among women with a singleton cephalic fetus.

## Materials & Methods

The meta-analysis was conducted based on the Cochrane Collaboration and the Preferred Reporting Items for Systematic Review and Meta-analysis (PRISMA) guidelines (File S1) (Page et al., 2021). The protocol of this study was pre-registered on the International Platform of Registered Systematic Review and Meta-analysis Protocols database under the number INPLASY2024120113. Clinical trial number: not applicable. All key processes (search, literature screening, data extraction, risk of bias assessment, and quality of evidence assessment) were performed independently by two reviewers. Disagreements were resolved by consensus or consultation with a third reviewer (MYT).

### Search strategy

Two reviewers (MZ and YW) independently searched the Cochrane Library, EMBASE, MEDLINE (*via* PubMed), Scopus, and Web of Science from the inception to December 25, 2024 (actual search date for all databases) using the following terms “labor, obstetric”, “birthing ball”, “peanut ball”, and their expansions combined in algorithms (File S2). We prioritized MeSH terms combined with keywords for retrieval, and adjusted the search strategy according to each database’s characteristics. An updated search was performed on November 10, 2025. To increase the sensitivity of the search, outcome-related terms were excluded from the search strategy (Huang et al., 2022). The reference lists of the included studies and relevant systematic reviews were screened for any additional studies. We did not search grey literature or trial registries, as our focus was on high-quality published clinical studies to reduce heterogeneity.

### Study selection

Included studies met the following criteria: (1) P (Population): pregnant women with a singleton and cephalic fetus; (2) I (Intervention): application of birthing balls or peanut balls; (3) C (Comparison): no intervention with birthing balls or peanut balls; (4) O (Outcomes): labor pain, delivery duration, and mode of delivery; (5) S (study design): randomized controlled trials. Additionally, only studies written in English were included.

Using the above criteria, two independent investigators (MZ and YW) first imported all retrieved records into EndNote for duplicate removal. Subsequently, they scrutinized the titles and abstracts of the retrieved records to exclude obviously irrelevant studies. After that, two independent reviewers (QHL and FX) read the full articles for final inclusion.

### Data extraction

Two independent investigators (QHL and FX) extracted data on characteristics of the included studies (first author, year of publication, registration detail, location of the trials, and sample size), characteristics of the participants (mean age, parity, inclusion criteria, and exclusion criteria), characteristics of the interventions (intervention, control, the protocol for birthing balls and peanut balls, timing of intervention, and use of analgesic), and outcomes measured. As the missing data has not been peer-reviewed, we did not contact the authors to provide it (Huang et al., 2021).

### Risk of bias assessment

Utilizing the Cochrane Collaboration’s tool (RoB 2.0), two reviewers (QHL and LYW) separately performed the risk of bias evaluation for the included studies (Sterne et al., 2019). High risk, low risk, or some concerns were used for the classification of the included studies.

### Data analysis

The synthesis of data was conducted using Stata 13.1 (StataCorp LP, College Station, TX). *p* < 0.05 was regarded as statistically significant. The main outcomes were labor pain and duration of delivery, whereas the additional outcome was mode of delivery. Considering the different mechanisms of action and types of exercise, birthing balls and peanut balls were analyzed separately, to minimize the clinical heterogeneity. For continuous variables, the mean difference (MD) with 95% confidence interval (CI) was calculated and standardized mean difference (SMD) alongside 95% CI was used where different methods were used to measure the same outcome. For dichotomous outcomes, risk ratio (RR) alongside 95% CI was adopted. The Cochrane Q statistics and I^2^ were used for the assessment of heterogeneity, and I^2^ > 50% was regarded as significant heterogeneity; thus, a random-effects model was adopted, otherwise, a fixed-effects model was chosen. Publication bias was assessed using funnel plots and Egger’s test in case at least ten studies are available for a certain outcome. Subgroup analyses were performed based on parity (nulliparous, primiparous, or multiparous), use of analgesic, and timing of intervention (initiated during laboring or pregnancy) when comparable data were available from at least two studies. Leave-one-out sensitivity analyses (*i.e.,* sequentially excluding each individual study and re-synthesizing data) were performed to assess the robustness of the results for each outcome.

### Grading the quality of evidence

Two investigators (QHL and FX) separately assessed the quality of the evidence for each outcome according to the Grading of Recommendations Assessment, Development, and Evaluation (GRADE) (Guyatt et al., 2011).

## Results

### Study selection

**Figure 1:**
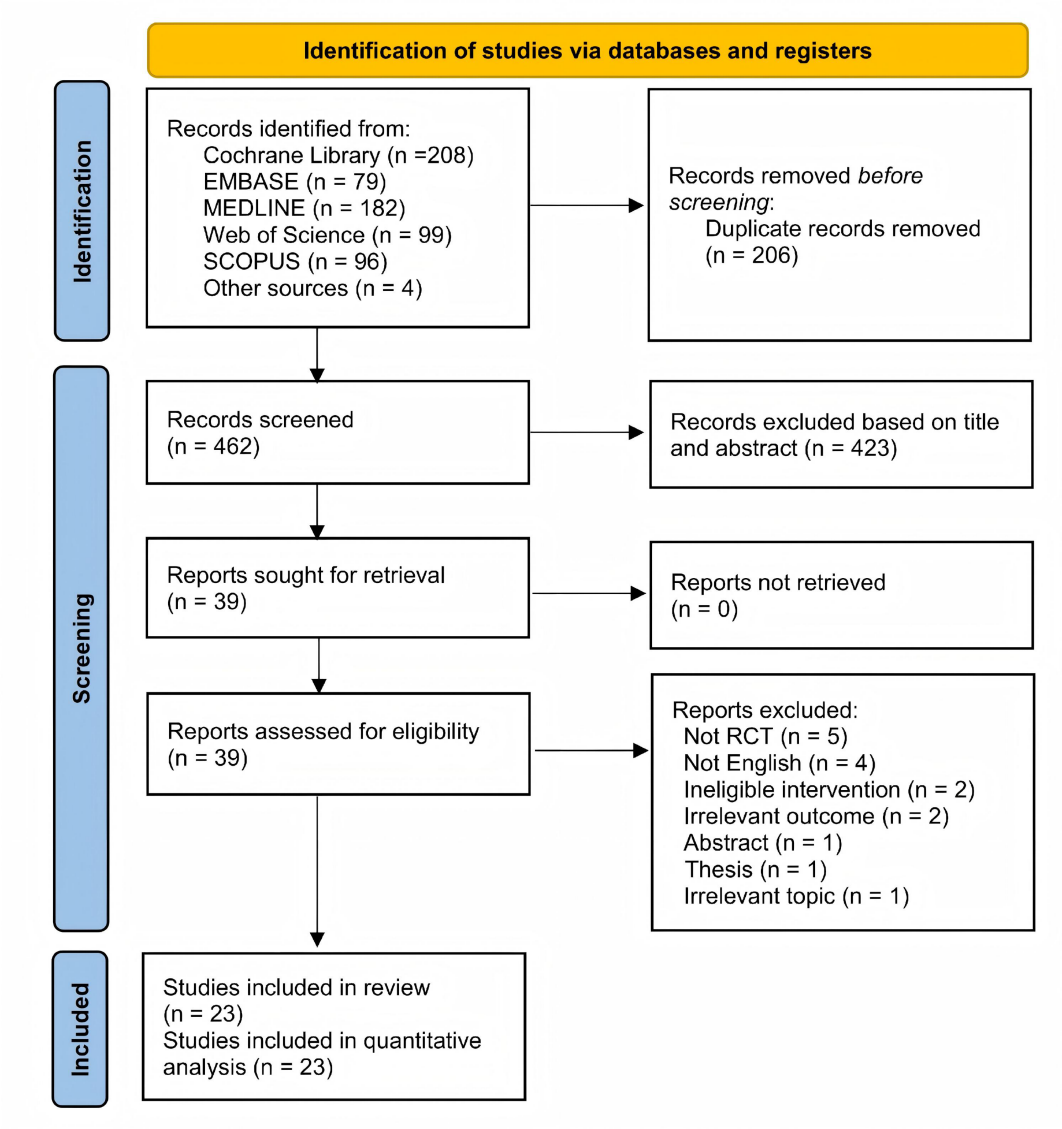
PRISMA flow diagram.

The flow diagram for search strategy and study selection process is illustrated in Fig. 1. A total of 668 potentially eligible records were initially retrieved, of which 208 from Cochrane Library, 79 from EMBASE, 182 from MEDLINE, 99 from Web of Science, 96 from Scopus, and four from other sources. Following the removal of 206 duplicate records using Endnote software, the title-abstract screening of the 462 remaining records led to 423 obviously irrelevant studies being eliminated. A full-text screening of the remaining 39 studies resulted in 16 studies being excluded because of various reasons. The list of excluded studies with reasons for exclusion is provided in File S3. As such, a total of 23 studies were included in this study, all of which were included in the quantitative analysis (Delgado et al., 2024; De Sena Fraga et al., 2024; Erkal Aksoy, Dereli Yilmaz & Çelimli, 2024; Gau et al., 2011; Taavoni et al., 2011; Mercier & Kwan, 2018; Roth et al., 2016; Alan Dikmen, Gönenç & Ataş, 2024; Mylod et al., 2024; Aktas et al., 2021; Vaijayanthimala & Arulappan, 2014; Aslantaş & Çankaya, 2023; Çankaya, Alan Dikmen & Ataş, 2025; Cavalcanti et al., 2019; Dunmez & Yilmaz, 2023; Gallo et al., 2014; Gallo et al., 2018; Mathew, Nayak & Vandana, 2012; Shen et al., 2021; Shirazi et al., 2019; Sönmez & Ejder Apay, 2023; Vaijayanthimala & Arulappan, 2014; Wang et al., 2020).

### Characteristics of the included studies

The characteristics of the included studies are shown in Table S1. The year of publication spanned from 2,011 to 2025. Overall, 3,192 participants were randomized to treatment, with a sample size of the studies included ranging from 40 to 294 and a mean age of 26.8 years. Most studies (18/23) enrolled primiparous and/or multiparous pregnant women, whereas three studies included solely nulliparous pregnant women and two studies did not report maternal parity information. Sixteen studies investigated the efficacy of birthing balls, six focused on peanut balls, and one study examined both birthing balls and peanut balls. Variations in the protocol of the intervention and the timing of intervention were found in the included studies. Four of the included studies utilized the birthing ball to practice exercise at home during pregnancy (Gau et al., 2011; Mylod et al., 2024; Aktas et al., 2021; Shirazi et al., 2019), while the other 19 studies performed exercises on birthing balls or peanut balls in various positions only in labor. For birthing ball exercises, sitting, standing, kneeling, and squatting were the commonly used positions, whereas side-lying and knee lateral rotation positions were adopted in peanut ball trials. Active phase of labor was the most common timing of intervention. Use of an epidural analgesic was permitted in three out of seven peanut ball trials, while two trials (De Sena Fraga et al., 2024; Alan Dikmen, Gönenç & Ataş, 2024) did not use it. However, six trials of birthing balls, one trial of peanut balls, and one trial assessing both birthing and peanut balls did not report on whether analgesia was used or not during labor. Labor pain, duration of the first, second, and/or third stages, and mode of delivery were universally reported in the included studies.

### Risk of bias assessment

**Figure 2:**
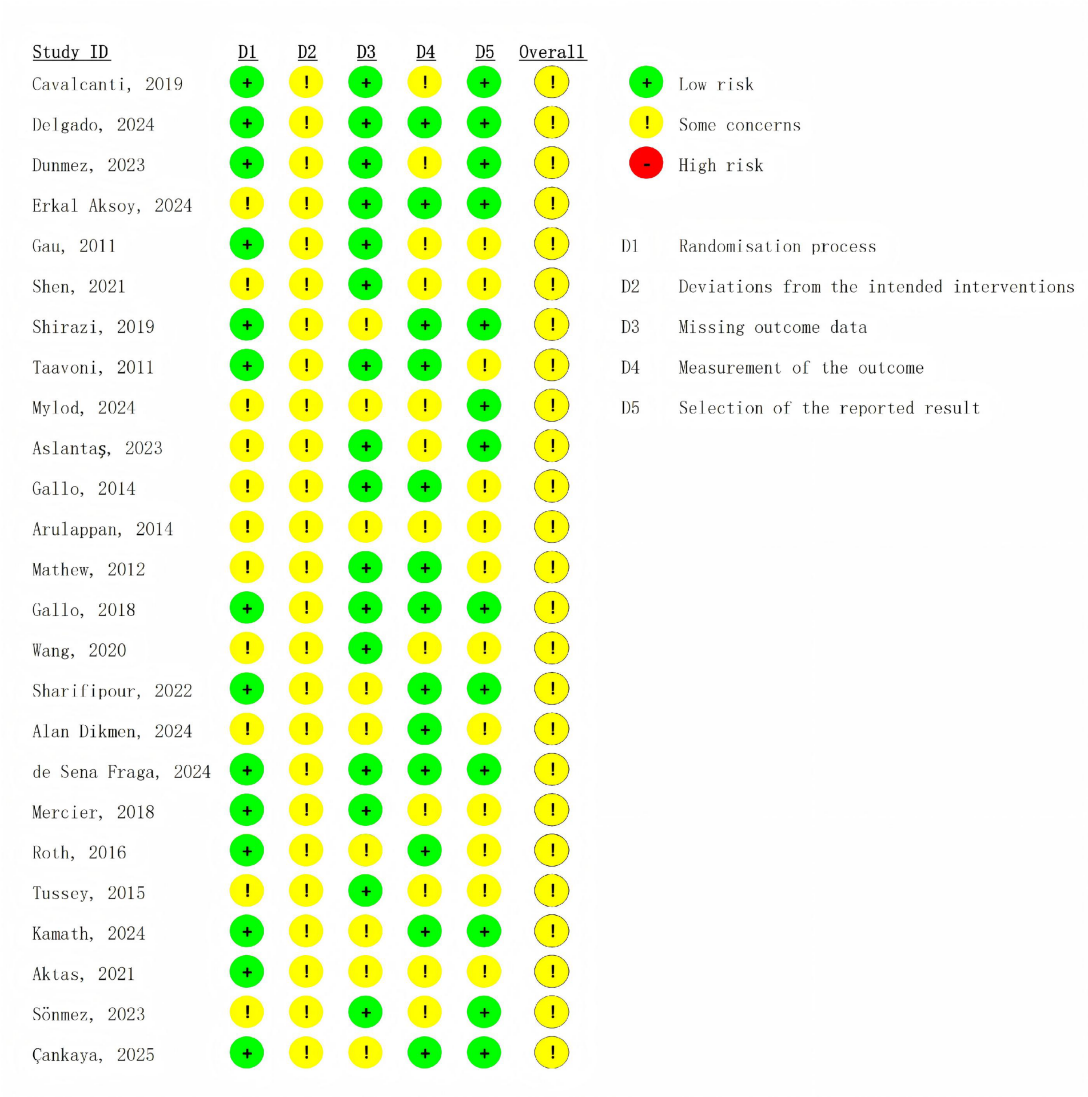
Risk of bias graph of the included studies.

Figure 2 shows the risk of bias of the included studies. Overall, all of the included studies were graded as having some concerns. Specifically, 47.8% (11/23) of studies were judged to have some risk of bias for the randomization process, mainly due to the insufficient description of random sequence concealment. Additionally, all studies were determined to have some risk of bias for deviations from the intended interventions, as due to the nature of the intervention, it was impossible to blind the participants. 30.4% (7/23) of studies showed some risk of bias due to more than 5% of participants being lost after randomization. 52.1% (12/23) of studies were classified as having some concerns in outcome measurement, owing to either explicit non-blinding of outcome assessors or insufficient description of their blinding status. Regarding the selection of the reported result, 52.1% (12/23) of studies were restricted by the missing pre-registration information and, therefore, were rated as having some concerns.

### Meta-analysis 1: efficacy of birthing balls for labor and delivery

#### Labor pain

**Figure 3:**
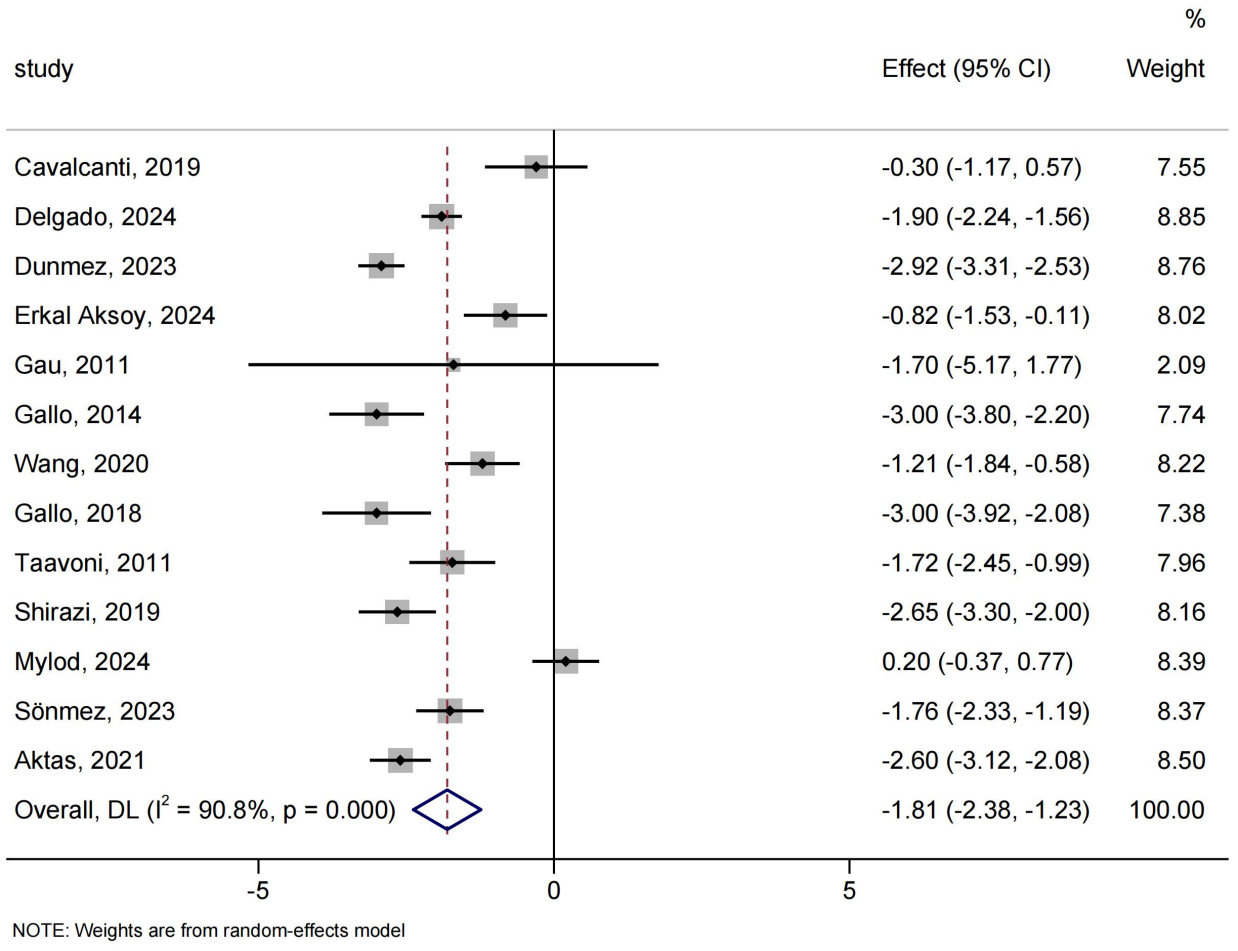
Forest analyses of the efficacy of birthing balls for labor pain. Effects are expressed as mean difference with 95% CI.

Labor pain measured by visual analog scale or numeric categorical scale was reported in 13 out of 17 trials, involving 1,325 participants. Meta-analysis showed that birthing balls significantly reduced labor pain relative to no birthing balls (MD: −1.81; 95% CI [−2.38 to −1.23]; *I*^2^ = 90.8%; Fig. 3). Although the overall heterogeneity was substantial and could not be fully explained by subgroup analyses, the findings remained robust in the leave-one-out sensitivity analyses (File S4 and S5).

#### Duration of labor

**Figure 4:**
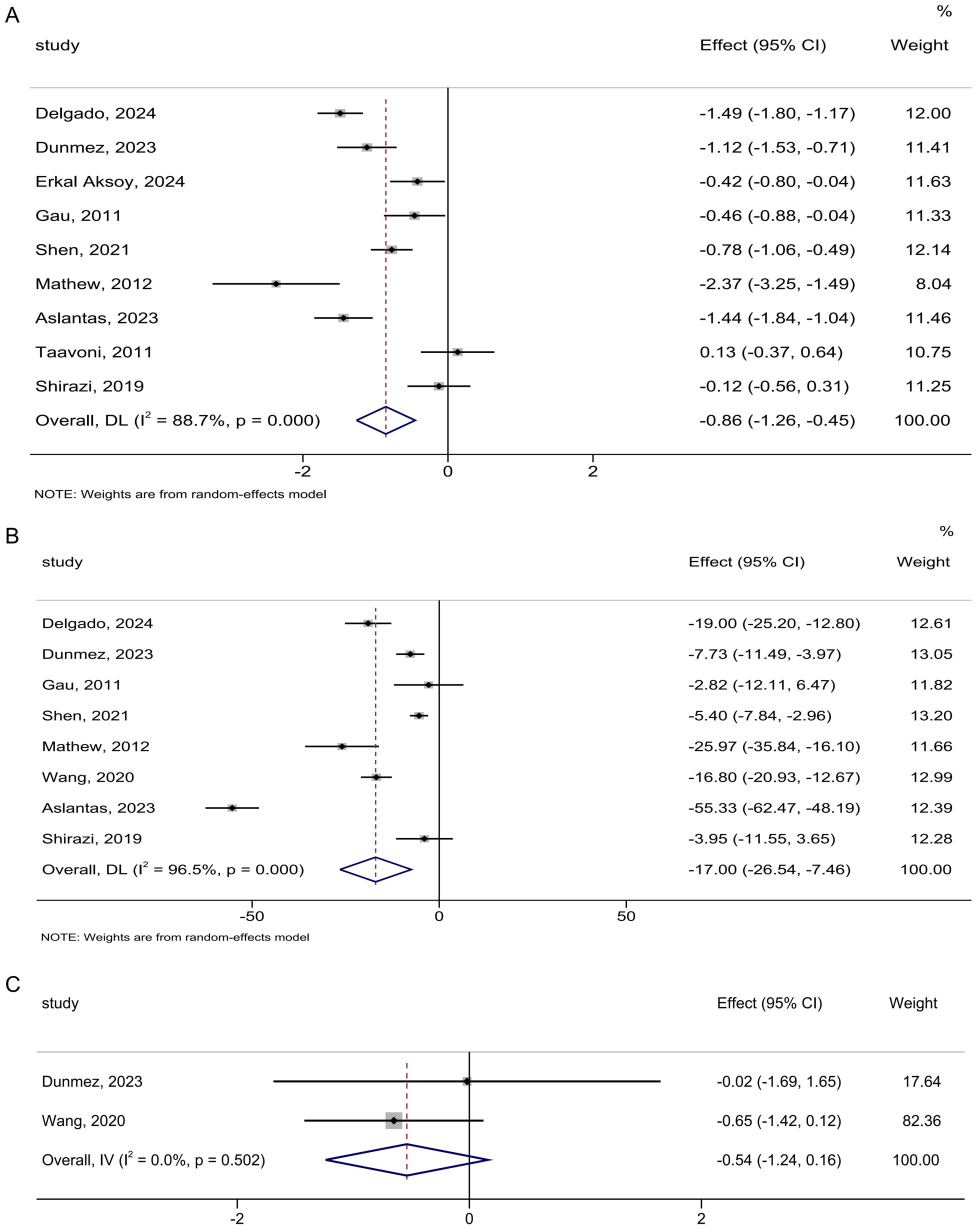
Forest analyses of the efficacy of birthing balls for (A) duration of the first stage of labor, (B) duration of the second stage of labor, and (C) duration of the third stage of labor. Effects are expressed as mean difference with 95% CI for (B and C), standardized mean difference with 95% CI for (A).

The duration of the first stage of labor included nine studies comprising 1,005 participants. Meta-analysis showed that birthing balls were associated with more efficacious than no birthing balls in shortening the duration of the first stage of labor (SMD: −0.86; 95% CI [−1.26 to −0.45]; *I*^2^ = 88.7%; Fig. 4A). Leave-one-out sensitivity analyses did not make significant alterations in the overall findings (File S4).

The duration of the second stage of labor involved eight studies comprising 944 participants. Meta-analysis showed that birthing balls significantly reduced the duration of the second stage of labor compared to no birthing balls (MD: −17.00 min; 95% CI [−26.54 to −7.46]; *I*^2^ = 96.5%; Fig. 4B). The overall findings did not differ following omitting any study of the studies included (File S4).

Two studies involving 216 participants were included in the analysis of the duration of the third stage of labor, and the result showed that there was no significant difference between birthing balls and no birthing balls (MD: −0.54 min; 95% CI [−1.24 to 0.16]; *I*^2^ = 0.0%; Fig. 4C). Leave-one-out sensitivity analyses did not significantly change the overall findings (File S4).

#### Mode of delivery

**Figure 5:**
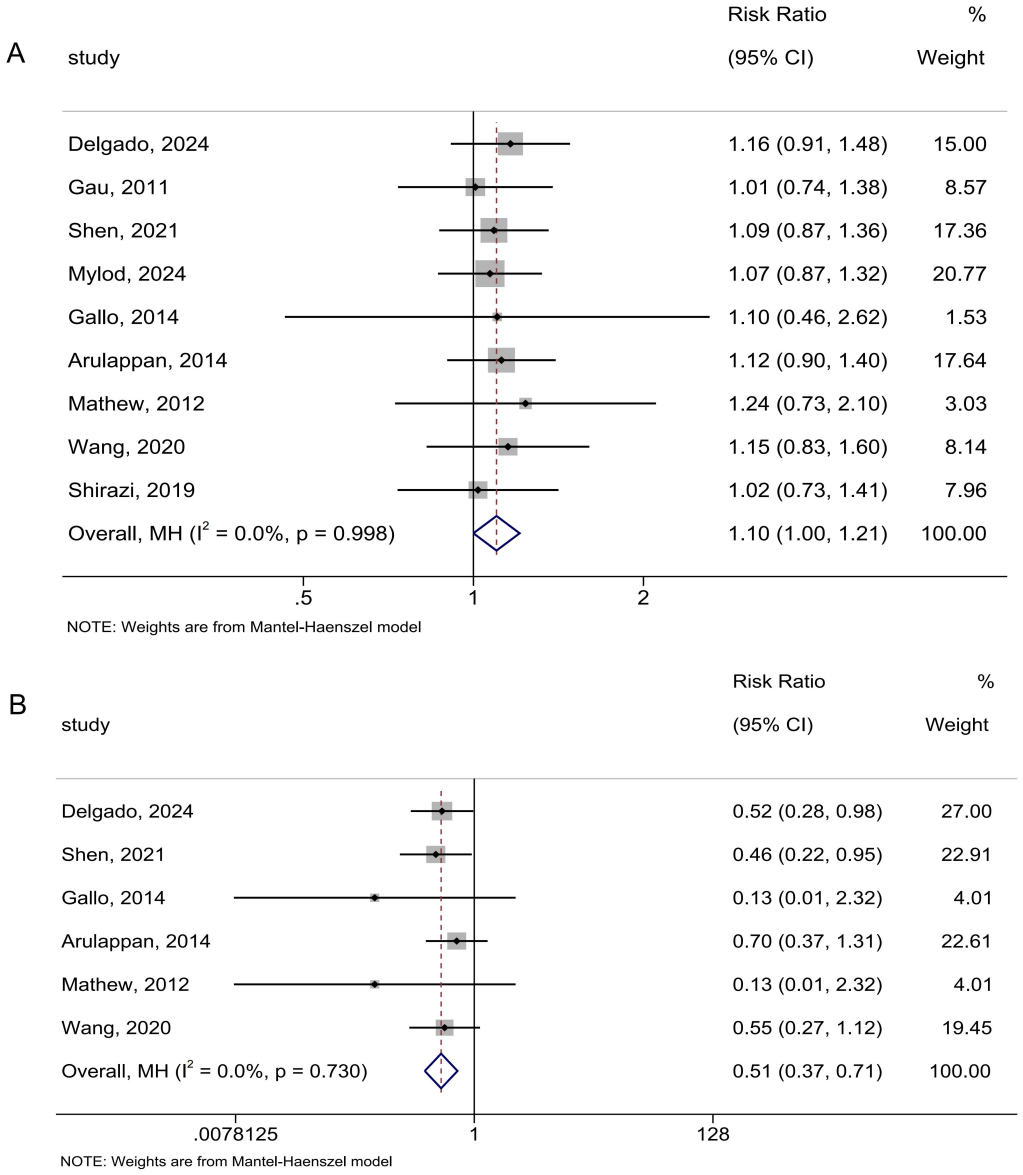
Forest analyses of the efficacy of birthing balls for (A) chance of vaginal delivery and (B) incidence of cesarean delivery. Effects are expressed as risk ratio with 95% CI for (A and B).

In the analysis of spontaneous vaginal delivery, there were nine studies involving 1,251 participants. The birthing ball was not found to have better efficacy in increasing the chance of spontaneous vaginal delivery than no birthing ball (RR: 1.10; 95% CI [1.00–1.21]; *I*^2^ = 0.0%; Fig. 5A). Omitting the study by Gau et al. (2011) or Shirazi et al. (2019) rendered the result significant (File S4).

In the analysis of cesarean delivery, there were six studies including 801 participants. The birthing ball was found to better reduce the incidence of cesarean delivery than no birthing ball (RR: 0.51; 95% CI [0.37–0.71]; *I*^2^ = 0.0%; Fig. 5B). The overall findings persisted in the leave-one-out sensitivity analyses (File S4).

### Meta-analysis 2: efficacy of peanut balls for labor and delivery

#### Labor pain

**Figure 6:**
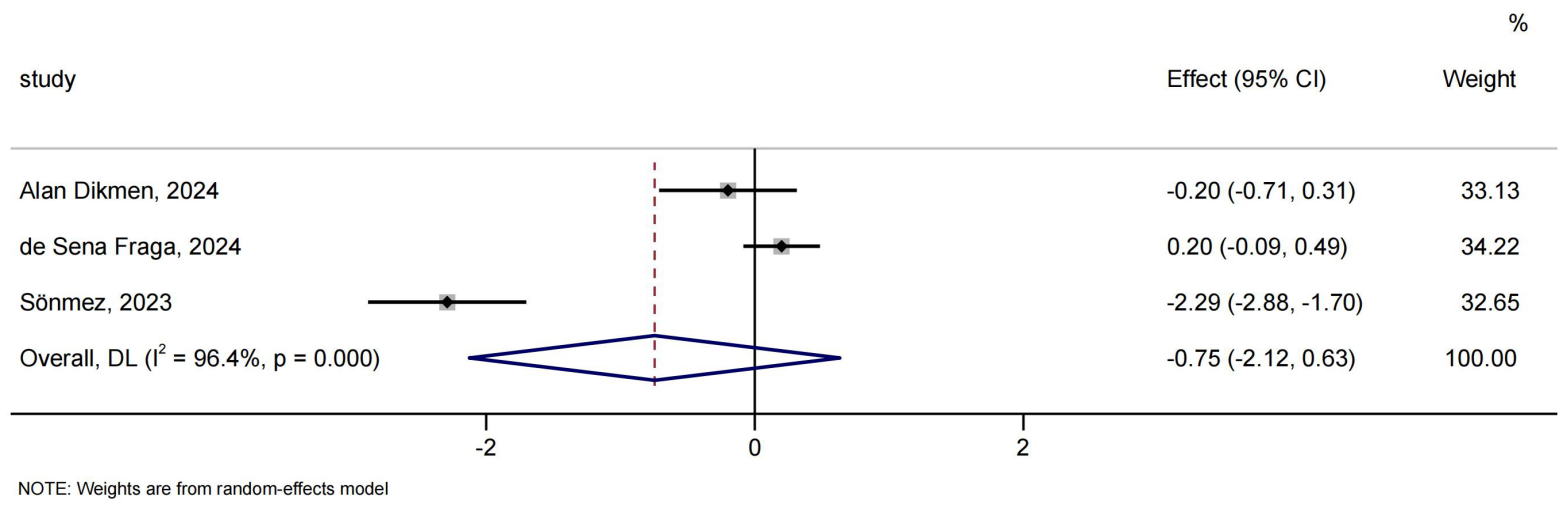
Forest analyses of the efficacy of peanut balls for labor pain. Effects are expressed as mean difference with 95% CI.

Three studies comprising 310 participants were included in the analysis of labor pain. The meta-analysis result showed that there was no significant difference between peanut balls and no peanut balls in alleviating labor pain (MD: 0.75; 95% CI [−2.12 to 0.63]; *I*^2^ = 96.4%; Fig. 6). Omitting any studies did not significantly alter the overall findings (File S4).

#### Duration of labor

**Figure 7:**
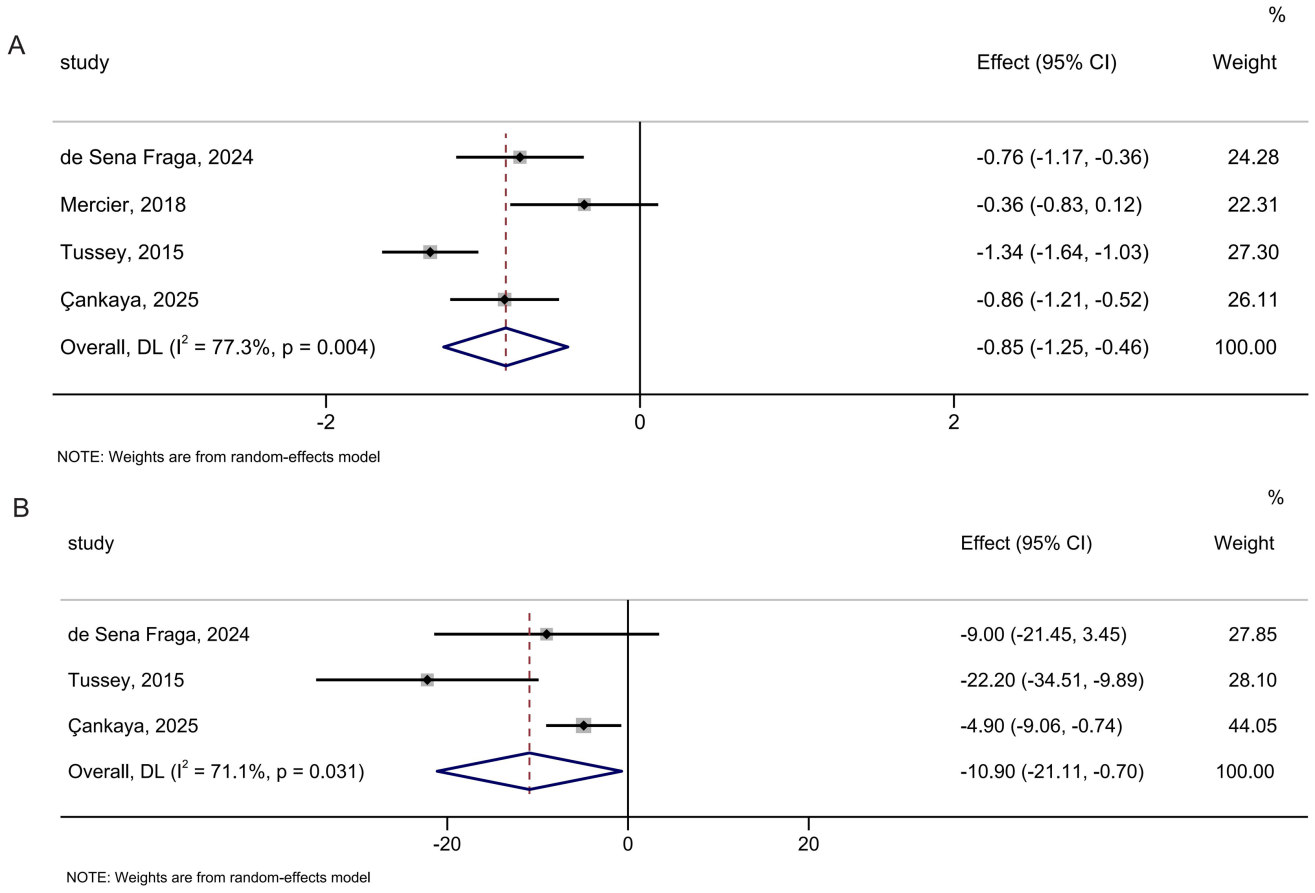
Forest analyses of the efficacy of peanut balls for (A) duration of the first stage of labor and (B) duration of the second stage of labor. Effects are expressed as standardized mean difference with 95% CI for (A) and mean difference with 95% CI for (B).

In the analysis of the duration of the first stage of labor, four studies involving 512 participants were included. Peanut ball was found to significantly shorten the duration of the first stage of labor compared to no peanut balls (SMD: −0.85; 95% CI [−1.25 to −0.46]; *I*^2^ = 77.3%; Fig. 7A). The overall findings remained robust in the leave-one-out sensitivity analyses (File S4).

In the analysis of the duration of the second stage of labor, three studies involving 431 participants were included. Peanut ball was found to significantly shorten the duration of the second stage of labor relative to no peanut ball (MD: −10.90 min; 95% CI [−21.11 to −0.70]; *I*^2^ = 71.1%; Fig. 7B). Omitting the study by De Sena Fraga et al. (2024) made the result not significant (File S4).

#### Mode of delivery

**Figure 8:**
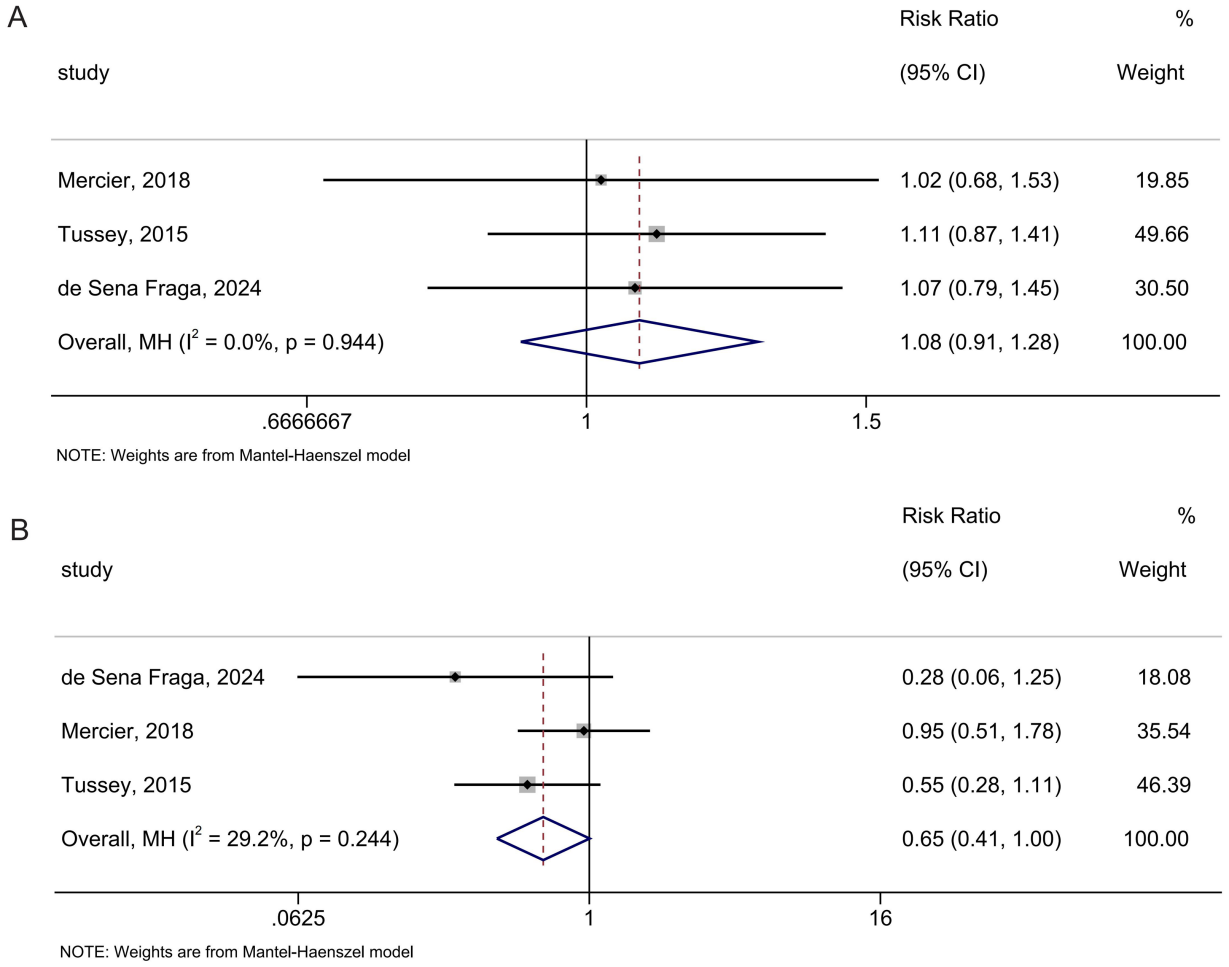
Forest analyses of the efficacy of peanut balls for (A) chance of vaginal delivery and (B) incidence of cesarean delivery. Effects are expressed as mean difference with 95% CI for risk ratio with 95% CI.

In the analysis of spontaneous vaginal delivery, three studies involving 387 participants were included. Peanut ball was not found to have better efficacy in increasing the incidence of spontaneous vaginal delivery than no peanut ball (RR: 1.08; 95% CI [0.91–1.28]; *I*^2^ = 0.0%; Fig. 8A). The overall findings remained in the leave-one-out sensitivity analyses (File S4).

For cesarean delivery, there were three studies including 387 participants in the meta-analysis. Peanut ball was found not to better reduce the incidence of cesarean delivery than no peanut ball (RR: 0.65; 95% CI [0.41–1.00]; *I*^2^ = 29.2%; Fig. 8B). Omitting the study by Mercier & Kwan (2018) made the result significant (File S4).

### Subgroup analysis

The insufficient quantity and information of the included studies prevented reliable analysis of any predefined subgroup analysis, except subgroup analysis based on the timing of intervention and use of analgesic.

For subgroup analysis based on intervention timing, stratified analyses revealed that initiating birthing ball exercises during active labor (intrapartum), compared to no birthing ball use, was associated with greater efficacy in shortening the first stage of labor (SMD: −1.02; 95% CI [−1.48 to −0.56]; *I*^2^ = 88.9%, p for subgroup difference = 0.012) and the second stage of labor (MD: −21.36 min; 95% CI [−32.88 to −9.84]; *I*^2^ = 97.4%, p for subgroup difference = 0.007); for labor pain relief, although one subgroup (during labor) showed a statistically significant benefit (MD: −1.86; 95% CI [−2.42 to −1.29]; *I*^2^ = 87.7%) and the other (during pregnancy) did not (MD: −1.68; 95% CI [−3.39 to 0.02]; *I*^2^ = 95.2%), the between-group difference was not statistically significant (p for subgroup difference = 0.852), indicating only a potential trend rather than a definitive subgroup effect (File S5). In contrast, antenatal use of birthing balls (during pregnancy, prior to labor onset) showed no significant effects on these outcomes, and the heterogeneity was substantially reduced within this subgroup. This suggests that the timing of birthing ball use may partially account for the overall heterogeneity observed.

For subgroup analysis based on analgesic use, stratified analysis showed that peanut balls significantly reduced the duration of the first stage of labor in women who did not use analgesia (SMD: −0.82; 95% CI [−1.09 to −0.56]; I^2^ = 0%), but had no effect in those who received epidural analgesia (SMD: −0.86; 95% CI [−1.82 to 0.10]; *I*^2^ = 91.4%). However, there was no statistical significance between the two subgroups (p for subgroup difference = 0.935; File S5).

### Publication bias

Publication bias was not found with funnel plots and Egger’s test (*p* = 0.55) for labor pain in the meta-analysis of birthing balls (File S6). Other outcomes had insufficient available studies to induce publication bias assessment.

### Quality of evidence assessment

Quality of evidence assessment for each outcome is shown in File S7. Overall, low- to moderate-quality evidence was observed for birthing balls and peanut balls. Briefly, all outcome was downgraded due to unclear risk of bias within study, and in terms of imprecision, down-gradation was applied to five outcomes owing to the small sample size and one outcome for birthing balls due to confidence intervals including values favoring either treatment.

## Discussion

Overall, we found low- to moderate-quality evidence favored the initiation of birthing balls exercises during laboring (but not during pregnancy) in relieving labor pain and shortening the duration of the first and second stage s of labor, thus reducing the incidence of cesarean delivery, whereas the application of peanut balls was supported only in the shortening of the duration of the first and second stages of labor, both as effective non-pharmacological interventions. Of note, these findings remained in the majority of leave-one-out sensitivity analyses, especially in the analyses of the efficacy of birthing balls, which may partly prove the robustness of this meta-analysis. However, the scarce number of studies on peanut balls prevented further analysis and therefore more well-designed studies are needed. In addition, limited by the universal unclear risk of bias in the included studies, the present findings must be interpreted with caution.

Previous reviews have paid attention to the efficacy of birthing balls (Grenvik et al., 2022) and peanut balls (Ahmadpour et al., 2021; Delgado et al., 2022). Consistent with our findings, the aforementioned reviews by Grenvik et al. (2022) and Delgado et al. (2022) also supported the use of birthing balls to alleviate labor pain and the use of peanut balls to shorten the duration of the first stage of labor. However, the previous reviews were limited by the number and quality of the included studies, in which non-randomized controlled trials or conference abstracts were permitted to be included in the review. It is therefore unclear whether the statistical power is too small to detect the effectiveness or whether indeed there are no therapeutic effects on other outcomes. Additionally, the efficacy of peanut balls for labor pain in women without an epidural is still unknown. A previous review also called for further research to determine their true effects (Grenvik et al., 2019). Up to now, more randomized controlled trials have been published since then. As such, an updated meta-analysis on birthing balls and peanut balls including studies to date is crucial to answer these questions.

While previous reviews did not support the efficacy of birthing balls on the duration of delivery and peanut balls on the second stage of labor (Grenvik et al., 2019; Delgado et al., 2022; Grenvik et al., 2022), our study offered new insights. First, our results favored the use of birthing balls to shorten the length of the first and second periods of labor, increase the chance of spontaneous vaginal delivery, and reduce the incidence of cesarean delivery. Second, the use of peanut balls could not relieve labor pain in laboring women. Third, the use of peanut balls could shorten the duration of the second stage of labor. One explanation might be attributed to the inclusion of more randomized controlled trials, which might improve the statistical power and have the ability to determine the true effects of birthing balls and peanut balls for more outcomes related to labor and delivery. Specifically, the search of the most recent reviews was dated to 2020, and the updated search resulted in 14 new trials being included in this meta-analysis (Delgado et al., 2024; De Sena Fraga et al., 2024; Erkal Aksoy, Dereli Yilmaz & Çelimli, 2024; Alan Dikmen, Gönenç & Ataş, 2024; Mylod et al., 2024; Aktas et al., 2021; Aslantaş & Çankaya, 2023; Çankaya, Alan Dikmen & Ataş, 2025; Cavalcanti et al., 2019; Dunmez & Yilmaz, 2023; Shen et al., 2021; Shirazi et al., 2019; Sönmez & Ejder Apay, 2023; Wang et al., 2020). Another reason might be the exclusion of conference abstracts and low-quality studies, which might help us obtain more accurate results and improve our confidence in the evidence. Another explanation that must be considered is that birthing ball exercises involving pelvic biomechanics or special pelvic movements were adopted in recent studies (Delgado et al., 2024; Erkal Aksoy, Dereli Yilmaz & Çelimli, 2024; Dunmez & Yilmaz, 2023; Sharifipour et al., 2022), rather than solely bouncing on the ball, which may better widen the pelvis, facilitate fetal descent and rotation and thus shorten the duration of the first and second periods of labor, increase the likelihood of spontaneous vaginal delivery, and reduce the risk of cesarean delivery.

The unique shape of the peanut ball determines that it can be used in women with an epidural. However, the use of peanut balls under the influence of an epidural does not reflect the reality of most women in the world and may hinder the evaluation of labor pain by peanut balls. Of note, the effect of peanut balls on labor pain was measured by two most recent studies without the use of an epidural, which ruled out the influence of analgesia on the determination of the effect of peanut balls on labor pain (De Sena Fraga et al., 2024; Alan Dikmen, Gönenç & Ataş, 2024). Additionally, pelvic mobility exercises (*e.g.*, rocking, rotating, or tilting the pelvis while seated on a ball) are of clinical significance, exerting therapeutic effects through physiological pain mechanisms, reducing the nociceptive response and helping to alleviate pain during contractions (Melzack & Wall, 1965; Smith et al., 2018a). These exercises were integrated into most birthing ball interventions across the included studies. In contrast, pelvic mobility exercises were adopted only in three out of the seven included studies of peanut balls (De Sena Fraga et al., 2024; Çankaya, Alan Dikmen & Ataş, 2025; Sönmez & Ejder Apay, 2023), while the other four studies solely placed peanut balls between legs in various positions. This discrepancy in intervention design—specifically the lack of pelvic mobility in peanut ball applications—may explain why birthing balls demonstrated superior efficacy in labor pain relief compared to peanut balls in our analysis. Notably, this aligns with our subgroup analysis: peanut balls reduced first-stage labor duration in non-analgesia users (able to perform pelvic mobility) but not in epidural users (mobility-restricted), reflecting a trend that pelvic mobility modulates intervention effectiveness.

The strong point of this meta-analysis lies in the inclusion of the most relevant randomized controlled trials, which helps with decision-making during labor and can be used as a tool to identify areas in which research is still insufficient. Additionally, only randomized controlled trials were allowed to be included in the meta-analysis, and RoB 2.0 and the GRADE tool were utilized to assess the overall quality of evidence for each outcome, which helped us obtain more objective and accurate conclusions. Furthermore, due to the difference in mechanisms of action and components of intervention, birthing balls and peanut balls were analyzed separately, to increase the clinical homogeneity. Finally, stratified analyses based on the timing of intervention were performed and thus more specific effects can be determined. In addition, we performed sensitivity analyses to assess the robustness of the findings, and applied the GRADE approach to objectively evaluate the potential impact of bias on the overall certainty of evidence.

This meta-analysis inevitably has some limitations. Though 14 new trials were included in this study, some outcomes, especially those for peanut balls, were limited by the small number of studies; therefore, subgroup analyses based on parity and use of analgesics could not be performed. Additionally, some concerns were raised when evaluating the within-study bias and therefore the certainty of evidence was downgraded by one level. The certainty of evidence was also downgraded by one level due to the small sample size and confidence interval including values favoring either treatment. As a result, the findings of this study were based on low- to moderate-quality evidence. Moreover, the language was restricted to English, leaving studies published in other languages might not to be included. Furthermore, the research data sources did not cover all recommended primary study databases, including additional subject-specific databases, citation indexes, trial registers, and unpublished/ongoing studies. Finally, potential COVID-19 impacts (*e.g.*, modified clinical practice, limited maternal activity) cannot be ruled out, yet subgroup analysis by pandemic period was hindered by incomplete temporal data of the included studies.

Future studies should therefore design more rigorous randomized controlled trials, particularly in terms of the concealment of the randomization process and the blinding of outcome assessors. Additionally, stratified data on parity (nulliparous, primiparous, or multiparous) and detailed duration of the first stage of labor (active and transition) are encouraged to be reported in future studies, to facilitate systematic reviews and thus allow more specific conclusions. Moreover, in view of the potential effect of peanut balls on the duration of labor, the wider range of applications, and the small number of studies available, more studies investigating the efficacy of peanut balls on labor and delivery are needed. In the end, directly comparing peanut balls and birth balls may deepen our understanding of their therapeutic effects and mechanisms of action.

## Conclusions

Based on low- to moderate-quality evidence, birthing balls may be considered a potentially effective tool for alleviating labor pain and shortening the duration of the first and second stages of labor, thus reducing the likelihood of cesarean delivery; peanut balls may also shorten the duration of the first and second stages of labor. Given the limitations in study quality and the lack of information in the available studies, more well-designed studies are needed.

##  Supplemental Information

10.7717/peerj.21062/supp-1 Supplemental Information 1 Characteristics of included studies (N = 23)

10.7717/peerj.21062/supp-2 Supplemental Information 2 PRISMA checklist

10.7717/peerj.21062/supp-3 Supplemental Information 3 Search strategy

10.7717/peerj.21062/supp-4 Supplemental Information 4 List of excluded studies with reasons for exclusion

10.7717/peerj.21062/supp-5 Supplemental Information 5 Leave-one-out sensitivity analyses

10.7717/peerj.21062/supp-6 Supplemental Information 6 Subgroup analyses of birthing balls based on timing of intervention

10.7717/peerj.21062/supp-7 Supplemental Information 7 Funnel plot for labor pain by birthing balls

10.7717/peerj.21062/supp-8 Supplemental Information 8 GRADE Profile of the Included Studies

10.7717/peerj.21062/supp-9 Supplemental Information 9 Systematic Review and/or Meta-Analysis Rationale

## Additional Information and Declarations

### Data Availability

The following information was supplied regarding data availability: This is a systematic review and meta-analysis.
