# Supplementary material for: Birthing balls and peanut balls for labor pain, delivery duration, and mode of delivery: a meta-analysis of randomized controlled trials
Source: PeerJ. 2026 Apr 2;14:e21062. doi: 10.7717/peerj.21062 (PMC13050517; doi:10.7717/peerj.21062)
Supplement: Supplemental Information 1 [file peerj-14-21062-s001.docx]

Table 1. Characteristics of included studies (N = 23)

| Study (First author, Year) | Trials | | Sample size | Participants | | | | Intervention | Control | Birthing /peanut ball Protocol | Timing of intervention | Use of analgesic | Outcome |
| --- | --- | --- | --- | --- | --- | --- | --- | --- | --- | --- | --- | --- | --- |
|  | Registration detail | Location of the trial |  | Age (years) | Parity | Inclusion criteria | Exclusion criteria |  |  |  |  |  |  |
| Cavalcanti, 2019 | Brazilian Registry of Clinical Trials (NO. 84 XPRT) | São Paulo, Brazil | Randomized:  EG: 39  CG: 44  Analyzed:  EG: 39  CG: 44 | EG: 24.56 (4.91)  CG: 26.05 (5.41) | Any | Active phase of labor; 2-3 uterine contractions in ten min; cervical dilation 3-8 cm; age gestational 37-42 complete weeks; ≥ 18 years old; single fetus; alive; pain ≥ 5 | Indication of cesarean delivery; with analgesia; smokers; disorders; psychoactive drug users; ingested caffeine in the last 10 h; ＜ 6 prenatal consultations; used synthetic or natural corticosteroids | Birthing ball | No birthing ball | Women sat on the ball with a flexed leg forming a 90º angle, with the knees apart and the plantar area of the feet resting on the ground, performing propulsion (low and rising) and pelvic rotation movements, for 30 min | Active phase of labor | No | VAS  VAS-anxiety |
| Delgado, 2024 | NCT04124835 | 3 in Recife and 1 in  Vitória de Santo Antão, Brazil | Randomized:  EG: 100  CG: 100  Analyzed:  EG: 100  CG: 100 | EG: 25 (4)  CG: 26 (3) | Any | Active phase of labor, low-risk full-term pregnancy and single fetus in cephalic presentation | Fetal death; indication for caesarean section; difficulty in remaining upright; use of psychoactive drugs; and epidural analgesia or oxytocin given  prior to randomisation | Birthing ball | No birthing ball | Women performed pelvic anteversion and retroversion, lateral pelvic tilts and circular hip movements according to individual obstetric evaluation (fetal station and position, cervical dilatation and the presence of early pushing urge) on a Swiss ball | Active phase of labor | No | Duration of labor, VAS, fatigue, anxiety, maternal satisfaction, perineal outcome, neonatal outcomes, oxytocin use, and epidural analgesia use |
| Dunmez, 2023 | NCT05360823 | Turkey | Randomized:  EG: 55  CG: 55  Analyzed:  EG: 53  CG: 53 | EG: 25.23 (3.92)  CG: 24.42 (3.61) | Primiparous | Primiparous, singleton pregnancy; aged 18-35; without risky pregnancy; age gestational 37-42; cervical dilation < 4 cm; would have a vaginal delivery; could speak Turkish | Nor reported | Birthing ball | No birthing ball | Women applied exercises on the birth ball (turning the hip completely, moving to the right/left, coming back and forth, and performing a slight jumping movement while sitting on the birth ball) when the contractions started and stopped when the contractions ended for an average of 25 min every hour | Active phase of labor | Not reported | VAS, Partograph follow-up, and Maternal Satisfaction Assessment at Delivery  Scale-Normal Birth |
| Erkal Aksoy, 2024 | NCT05312502 | Konya, Turkey | Randomized:  EG: 70  CG: 70  Analyzed:  EG: 57  CG: 54 | EG: 28.47 (5.98)  CG: 28.55 (4.68) | Primiparous  Multiparous | Over 18 years old; cervical dilation 4 cm; no complications preventing vaginal delivery; age gestational ≥ 36; singleton pregnancy; not risky pregnancy; without psychiatric problems | Emergency cesarean section during labor; narcotic analgesia applied; pregnancy complications | Birthing ball | No birthing ball | Women sit on the ball, pelvic rocking, forward–backward and right-left rocking, forward supported sitting, springing movements for a total of 30 min, and seated on the ball in an upright position during labor | Active phase of labor | No | VAS, Birth Process Follow-up From, and Birth Satisfaction Scale-Revised |
| Gau, 2011 | Not reported | Taiwan | Randomized:  EG: 94  CG: 94  Analyzed:  EG: 48  CG: 39 | EG: 30.1 (3.4)  CG: 30.3 (4.0) | Primiparous  Multiparous | 30–32 weeks of gestation; older than 18 years; no major obstetric or medical pregnancy complications; singleton pregnancy; normal extremities and ability; speak, read, and write Chinese | Admitted to the hospital prior to 37 weeks of gestation; estimated cervical dilations of more than 4 cm; used epidural anesthesia; underwent an emergency caesarean section | Birthing ball | No birthing ball | Women were asked to practice eight exercises program consisted of a 26-page booklet and a 19-minute videotape at home for at least 20 min three times per week for 6-8 weeks | 30-32 weeks of gestation | No | McGill Pain Questionnaire, Childbirth Self-Efficacy Inventory, and obstetrical information |
| Shen, 2021 | LYG-MEP2021005 in Clinical  Hospital centerSestre Milosrdnice  Trial registry | Lianyungang, China | Randomized:  EG: 100  CG: 100  Analyzed:  EG: 100  CG: 100 | EG: 27.7 (3.0)  CG: 28.0 (2.9) | Any | Single fetus, head  position, non-clinical, and reactive in the nonstress test of fetal heart rate monitoring; complete membranes, immature cervix, and Bishop score ≤ 6; amniotic fluid > 5.0 cm | Severe heart, lung, liver,  or kidney diseases; immune dysfunction; twin or multiple pregnancy; placenta previa; scarred uterus; reproductive tract infection. | Birthing ball | No birthing ball | Women sat on the ball holding a railing with both hands and gently swung the body for 30 min and could move freely on the delivery ball for 30 min when the occipital posterior position or occipital transverse position occurred during the second stage of labor | After 38 weeks of gestation | Not reported | Duration of each stage of labor, mode of delivery, cervical Bishop score, pH value of neonatal umbilical vein blood, partial PO_2_, partial PCO_2_, and the 1-min Apgar score |
| Mylod, 2024 | International Standard Randomised Controlled Trials (NO. 10755909) | UK | Randomized:  EG: 148  CG: 146  Analyzed:  EG: 148  CG: 146 | EG: 28.37  CG: 28.35 | Any | Nulliparous and parous; aged 18 and over; at low risk of obstetric intervention; singleton fetus and planned to labor and birth in hospital | Aged under 18; had a multiple pregnancy or planned to give birth either at home or with a planned caesarean section; had a history of caesarean section or other uterine surgery, cardiac, endocrine or obstetric complications, or used opiate analgesia or recreational drugs | Birthing ball | No birthing ball | Women practiced birthing ball exercise at home based on a bespoke online animated educational video entitled, “Having A Bill in Early Labor” | 36 weeks of gestation | Not reported | VAS, Expectancy and Self-efficacy Expectancy scores, cervical dilatation, birth mode, ball uptake, acceptability and maternal satisfaction |
| Aslantaş, 2023 | Not reported | Turkey | Randomized:  EG: 60  CG: 60  Analyzed:  EG: 60  CG: 60 | EG: 22.90 (3.11)  CG: 22.90 (2.90) | Primiparous | 18 years or older; 37–42 weeks of pregnancy; singleton pregnancy; cervical dilatation of 1–4 cm;  primiparous; not having any complications that prevent vaginal delivery; not having any extremity problems for using the birth ball and performing activities; and | Having maternal and fetal complications; getting pregnant by assisted reproductive techniques; electing cesarean section | Birthing ball | No birthing ball | Women performed exercises with the ball in 3 different positions: sitting, kneeling, and squatting for approximately 20 min for each movement and each movement was performed at least once | Active phase of labor | No | VAS, Personal information form, Childbirth Comfort Scale, Birth Outcomes  Information Form, and Mackey Childbirth Satisfaction Rating Scale |
| Gallo, 2014 | Not reported | São Paulo, Brazil | Randomized:  EG: 20  CG: 20  Analyzed:  EG: 20  CG: 20 | 19 (4) | Primiparous | Primiparous, literate, single fetus in cephalad position, low-risk pregnancy, 37 weeks of gestation, cervical dilatation 4-5 cm; with adequate uterine dynamics, with no use of drugs; intact membranes without associated risk factors; lack of cognitive or psychiatric problems | Admitted for labor induction; premature or early chorioamniorrhexis; not using uterotonic drugs before the active stage | Birthing ball | No birthing ball | Women performed pelvic mobility exercises with the ball, active pelvic anteversion and retroversion exercises, lateralization, circumduction and propulsion for 30 min | Active phase of labor | Not reported | Labor duration, dilatation and fetal descent speed, mode of delivery, Apgar score |
| Arulappan, 2014 | Not reported | Tamilnadu, India | Randomized:  EG: 120  CG: 120  Analyzed:  EG: 106  CG: 105 | Not reported | Primiparous | Primiparous at gestational age 37 to 40 weeks with initial cervical dilation ≥ 3 cm with single fetus with cephalic presentation and who had normal vitality | Parturient mothers who were receiving analgesics, induced by medical or surgical method, had rupture of membrane, had history of pregnancy and labor  complications, those who have medical complications like Diabetes mellitus, Asthma and Hypertension | Birthing ball | No birthing ball | Women practice the swaying movements and positions on birthing ball at least 20 minutes during their prenatal checkups | 36 weeks of gestation | Yes | Numerical rating scale, demographic information, Modified Labor Agentry, mode of delivery, duration of labor, contraction pattern, and use of oxytocin and analgesic |
| Mathew, 2012 | Not reported | Mangalore, India | Randomized:  EG: 20  CG: 20  Analyzed:  EG: 20  CG: 20 | Not reported | Nulliparous | Primigravida mothers in the first stage of  labor | Not reported | Birthing ball | No birthing ball | When uterine contraction occurred, woman rocked comfortably for 5–10 min | Cervical dilatation 1-3 cm | Not reported | Duration of labor, cervical dilatation rate, type of delivery, newborn outcome, cervicograph, and Apgar score |
| Gallo, 2018 | NCT01389128 | São Paulo, Brazil | Randomized:  EG: 40  CG: 40  Analyzed:  EG: 40  CG: 40 | EG: 21 (4)  CG: 22 (4) | Nulliparous | Primigravida parturient with a low-risk pregnancy; a gestational age > 37 weeks; a single fetus in the cephalic position; spontaneous onset of labor; cervical dilation of 4 to 5 cm and appropriate uterine contractions for this phase; intact ovular membranes; literacy, including the ability to understand the study | use of analgesic medications or other drugs that interfere with uterine motility from hospital admission until randomisation; cognitive or psychiatric problems; admission for induction of labor; premature rupture of chorioamniotic membranes; or other risk factors | Birthing ball | No birthing ball | Women performed pelvic mobility exercises on the Swiss ball including active pelvic anteversion, retroversion, lateralization, circumduction, and propulsion for 40 min when the cervix is 4-5 cm dilated | During the period of 4-5 cm of cervical dilation | Yes | VAS |
| Wang, 2020 | Not reported | Binzhou, China | Randomized:  EG: 55  CG: 55  Analyzed:  EG: 55  CG: 55 | EG: 36.8 (4.6)  CG: 36.9 (4.7) | Primiparous | Primipara, without fertility experience; single pregnancy; full-term pregnancy; having vaginal natural delivery indications | Patients with pregnancy complications, mental disorders, and cognitive impairment, or severe heart, brain and blood diseases | Birthing ball | No birthing ball | Women perform exercise with the ball, in sitting, kneeling, squatting, and prone positions | 3 cm dilation | Not reported | Delivery pain, mode of delivery, postpartum hemorrhage, duration of labor, General Comfort Questionnaire, anxiety, depression, and Labor Agentry Scale |
| Taavoni, 2011 | Not reported | Tehran, Iran | Randomized:  EG: 31  CG: 31  Analyzed:  EG: 29  CG: 31 | EG: 23.73 (4.07)  CG: 24.80 (3.30) | Primiparous | Primiparous women aged 18 to 35 with singleton pregnancies; cephalic presentation of fetuses; 38 to 40 complete weeks of gestation, anticipation of a normal birth, and no history of infertility | If there was a need for analgesic medication, or if obstetric complications occurred | Birthing ball | No birthing ball | Women sat on the ball and rock their hips back and forth or around in a circle for a minimum of 30 minutes | Active phase labor with 4-8 cm dilatation | No | VAS, duration of active phase of labor, and duration and interval of uterine contractions |
| Shirazi, 2019 | Iranian Registry of the Clinical Trials with the identical code of 20147 | Tehran, Iran | Randomized:  EG: 87  CG: 87  Analyzed:  EG: 43  CG: 39 | EG: 33.4 (4.3)  CG: 32.23 (3.9) | Primiparous  Multiparous | Having a tendency for participation; being in 30-32 weeks of gestation; having a normal pregnancy; having no history of a disease or obstetric complications based on a standard prenatal  chart | Being forced to receive extra medical intervention such as epidural analgesia or emergency termination of pregnancy, or undergoing a cesarean section | Birthing ball | No birthing ball | Women performed 4 types of position with eight exercises, including sitting (pelvic rocking—forward and back, Hula-Hula—side-to-side, and rocking), standing (leaning forward on the ball and leaning against the ball on the wall—up and down), kneeling (hugging the ball and pelvic rocking), and squatting (leaning against the ball on the wall) at least 20 min once, three time per weeks for 6-8 weeks at home | 30-32 weeks of gestation | No | VAS, childbirth self-efficacy inventory, and standard questionnaires |
| Aktas, 2021 | Not reported | Turkey | Randomized:  EG: 42  CG: 42  Analyzed:  EG: 30  CG: 30 | EG: 28.1 (2.7)  CG: 28.8 (2.9) | Primiparous | 18-35 years old, regular follow-up and prenatal check-ups, 35th week of gestation, singleton pregnancy, normal birth weight, vertex position, planned spontaneous vaginal delivery, cervical dilatation ≤2 cm, potential term birth | Gestational hypertension/diabetes mellitus, emergency cesarean section, fetal anomalies, antipsychotic use, hearing/communication disorders, physical disability, epidural anesthesia | Birthing ball | No birthing ball | Women performed ball exercises (appropriate-sized ball) during childbirth, with 8 exercises in 4 positions for 20 min at least 3 times in a week at home for approximately 6-8 weeks. They adopted the most comfortable positions and movements, practicing hourly. | 35th week | No | VAS |
| Alan Dikmen, 2024 | Not reported | Konya, Turkey | Randomized:  EG: 50  CG: 50  Analyzed:  EG: 45  CG: 45 | EG: 22.84 (2.72)  CG: 23.2 (3.47) | Not reported | Indication for vaginal delivery; cervical dilatation of 3 cm or more; 38–42 weeks of gestation; single, healthy, vertex positioned fetus, no complications; not using analgesia and anesthesia during the first and second stage of labor, not having  any physical disability; not having communication problems | Abnormal changes in fetal heart rate; an unexpected complication; high-risk pregnancy; cesarean section; use of forceps or vacuum; taking magnesium sulfate; signs of intrauterine infection; previous attendance at a maternity school or training on birth balls, and withdrawal from the study | Peanut ball | No peanut ball | The peanut ball was positioned between the pregnant woman’s legs and her back was supported with a pillow; five positions (side-lying, semi-sitting, supine, hand-knee, and squatting) were changed periodically, each position for 20 min with a10 min interval | 4 cm dilation | No | VAS, VAS-fatigue, Maternal Perception of Childbirth Scale, and personal information form |
| de Sena Fraga, 2024 | RBR-74wcnjc | Juazeiro, Brazil | Randomized:  EG: 50  CG: 50  Analyzed:  EG: 50  CG: 50 | EG: 25 (6)  CG: 24 (5) | Primiparous  Multiparous | Any parity; aged ≥ 18 years, in the active phase of labor  (cervical dilation ≥ 6 cm), with a single pregnancy, at ≥ 37 gestational weeks, with the fetus in cephalic presentation and at low gestational  risk | Any complication had occurred during labor or if they were admitted for labor induction at the time of enrolment into the study | Peanut ball | No peanut ball | Women performed postures according to the fetal location in the maternal pelvis (inlet/superior strait, midplane and outlet) and pelvic mobility until completion of the second stage of labor | ≥ 6 cm dilation | No | Duration of labor, numerical rating scale, childbirth fatigue questionnaire, mode of delivery, perineal laceration, use of oxytocin, Apgar score, and admission to intensive care unit and resuscitation |
| Mercier, 2018 | Not reported | Philadelphia, PA | Randomized:  EG: 44  CG: 46  Analyzed:  EG: 43  CG: 43 | EG: 28.2 (6.2)  CG: 26.7 (7.3) | Nulliparous | Nulliparous; singleton pregnancy in cephalic presentation; gestational age of 37.0 to 41.6 weeks, had cervical dilation < 6 cm; category 1 or category 2 fetal heart tracing acceptable for a trial of labor | Under 18 years of age, were non-English speaking, were known to have any major fetal anomalies, or had any contraindication to a trial of labor | Peanut ball | No peanut ball | Women used the peanut ball for a minimum of 15 minutes of each hour of subsequent labor until the full dilation reached | ≥ 6 cm dilation | Yes | Time of complete dilation, mode of delivery, cervical dilation, effacement, fetal station, and fetal  position |
| Roth, 2016 | Not reported | US | Randomized:  200  Analyzed:  EG: 78  CG: 71 | EG: 30.6 (4.3)  CG: 31.1 (4.5) | Primiparous  Multiparous | At least 18 years of age, were scheduled for an elective labor induction at or beyond 39 weeks gestation, and chose an epidural for pain management | No specific exclusion criteria except those implied by the inclusion criteria | Peanut ball | No peanut ball | Women placed the ball between their knees with rotation of lateral positions every 30 min or as indicated by patient/fetal status | Within 30 min after epidural placement | Yes | Duration of labor, method of delivery, and use of oxytocin |
| Tussey, 2015 | Not reported | US | Randomized:  EG: 107  CG: 94  Analyzed:  EG: 107  CG: 94 | EG: 27.5 (6.7)  CG: 27.3 (6.2) | Any | In active labor; using an epidural for pain control; with the fetus in the cephalic presentation; indication for either elective induction or augmentation of labor, such as mild preeclampsia, possible macrosomia, or patient request | Required magnesium sulfate for preeclampsia; had signs of an intrauterine infection; Category 3 fetal heart rate tracing | Peanut ball | No peanut ball | Women placed the ball between their legs until the cervix completely effaced and dilated and passive descent had occurred | Immediately after epidural placement (in active labor) | Yes | Duration of labor and mode of delivery |
| Sönmez, 2023 | NCT04827797 | Turkey | Randomized:  EG1: 60  EG2: 60  CG: 60  Analyzed:  EG1: 60  EG2: 60  CG: 60 | EG1: 30% 25 and older  EG2: 38.3% 25 and older  CG: 18% 25 and older | Not reported | ≥18 years old, term pregnancy (38–40 weeks), estimated fetal weight <4000g, normal pelvic diameter (vaginal examination), primiparous, singleton pregnancy, live fetus, cervical dilation 4 cm | Having risks regarding the pregnancy and the fetus, having a head presentation, and undergoing an emergency cesarean section | EG1: Birthing ball  EG2: Peanut ball | No birthing/peanut ball | EG1: Women performed movements (swing hips right-left, front-back, round the circle, jumping on the ball) in 4 different positions (sitting on a round birth ball leaning in front, kneeling on the floor and leaning on the birth ball, sitting on the ball, and side-lying position in bed)  EG2: Women performed movements (jumping, right-left, front-back on the ball) in five different positions (half-sitting position, tucked side-lying position, hands and knee fire hydrant position, straddling position, forward-leaning position) | Active phase in the first stage of labor (4 cm to 9 cm) dilation） | Not reported | VAS, personal information form, verbal rating scale, partograph, and The Scale for Measuring Maternal Satisfaction in Birth |
| Çankaya, 2025 | NCT06387680 | Turkey | Randomized:  EG: 78  CG: 78  Analyzed:  EG: 70  CG: 70 | EG: 23.6 (2.5)  CG: 23.4 (2.6) | Primiparous | Primiparous pregnant women with vaginal delivery indications, cervical dilation ≥5 cm, full-term pregnancy (38–42 weeks), singleton pregnancy, healthy vertex-position fetus | Dystocia-related complications, first-stage labor analgesia/anesthesia, physical limitations for study positions, communication disorders/insufficient Turkish proficiency, intrapartum abnormal fetal heart rate, unexpected pregnancy/fetal complications, high-risk pregnancy, magnesium sulfate use, intrauterine infection symptoms, prior antenatal classes, and birthing ball education history | Peanut ball | No peanut ball | Women placed the ball between their legs with back support by a pillow in five labor positions (left/right lateral, supine, semi-seated, hands-and-knees, squatting, and pushing), each position for 20 min followed by a 10-min break | 5 cm dilation | No | Duration of labor, Pregnant women’s personal information form, labor monitoring form, postpartum follow-up form, APGAR score, Birth memories and recall questionnaire, and Birth satisfaction scale-revised |

CG, control group; EG, experimental group; VAS, visual analog scale
