# Supplementary material for: Birthing balls and peanut balls for labor pain, delivery duration, and mode of delivery: a meta-analysis of randomized controlled trials
Source: PeerJ. 2026 Apr 2;14:e21062. doi: 10.7717/peerj.21062 (PMC13050517; doi:10.7717/peerj.21062)
Supplement: Supplemental Information 3 [file peerj-14-21062-s003.pdf]

Search strategy.

Pubmed (searched on 10/11/2025)

#1	labor, obstetric[MeSH Terms]	50,193
#2	(((((((labor) OR (labour)) OR (pregnancy)) OR (pregnant women)) OR (postpartum period)) OR (Obstetric delivery)) OR (Parturition)) OR (Obstetric labor)	3,754,742
#3	((((((((labor) OR (labour)) OR (pregnancy)) OR (pregnant women)) OR (postpartum period)) OR (Obstetric delivery)) OR (Parturition)) OR (Obstetric labor)) AND (((((((((((((((delivery ball) OR (childbirth ball)) OR (birth ball)) OR (birthing ball)) OR (swiss ball)) OR (exercise ball)) OR (sport ball)) OR (bobath ball)) OR (prana ball)) OR (pezzi ball)) OR (childbirth ball)) OR (peanut ball)) OR (peanutball)) OR (peanut labor ball)) OR (peanut shaped ball))	1,179
#4	random*	1,943,435
#5	#1 OR #2	3,754,742
#6	#3 AND #4 AND #5	182

Cochrane library (searched on 10/11/2025)

#1	MeSH descriptor: [Labor, Obstetric] explode all trees	3,165
#2	labor OR labour OR pregnancy OR pregnant women OR postpartum period OR Obstetric delivery OR Parturition OR Obstetric labor	103,132
#3	delivery ball OR childbirth ball OR birth ball OR birthing ball OR swiss ball OR exercise ball OR sport ball OR bobath ball OR prana ball OR pezzi ball OR childbirth ball OR	2,164

	peanut ball OR peanutball OR peanut labor ball OR peanut shaped ball	
#4	Random or Random Allocation or Randomized controlled trials	1,412,390
#5	#1 OR #2	103,171
#6	#3 AND #4 AND #5	208

EMBASE (searched on 10/11/2025)

#1	exp Labor, Obstetric/	43,421
#2	(labor or labour or pregnancy or pregnant women or postpartum period or Obstetric delivery or Parturition or Obstetric labor).af.	1,340,836
#3	(delivery ball or childbirth ball or birth ball or birthing ball or swiss ball or exercise ball or sport ball or bobath ball or prana ball or pezzi ball or childbirth ball or peanut ball or peanutball or peanut labor ball or peanut shaped ball).af.	396
#4	Random*	2,847,926
#5	#1 OR #2	1,341,070
#6	#3 AND #4 AND #5	79

Web of Science (searched on 10/11/2025)

#1	TS=(labor OR labour OR pregnancy OR pregnant women OR postpartum period OR Obstetric delivery OR Parturition OR Obstetric labor)	3,739,170
#2	TS=(delivery ball OR childbirth ball OR birth ball OR birthing ball OR swiss ball OR exercise ball OR sport ball OR bobath ball OR prana ball OR pezzi ball OR childbirth ball OR peanut ball OR peanutball OR peanut labor ball OR peanut shaped ball)	52,647
#3	TS=(random*)	4,468,059

#4	#1 AND #2 AND #3	99

Scopus (searched on 10/11/2025)

#1	TITLE-ABS-KEY ("labor" OR "labour" OR "pregnancy" OR "pregnant women" OR "postpartum period" OR "obstetric delivery" OR "parturition" OR "obstetric labor")	1,904,653
#2	TITLE-ABS-KEY ("delivery ball" OR "childbirth ball" OR "birth ball" OR "birthing ball" OR "swiss ball" OR "exercise ball" OR "sport ball" OR "bobath ball" OR "prana ball" OR "pezzi ball" OR "childbirth ball" OR "peanut ball" OR "peanutball" OR "peanut labor ball" OR "peanut shaped ball")	584
#3	ALL FIELDS (random*)	10,584,301
#4	#1 AND #2 AND #3	96
