# Supplementary material for: Birthing balls and peanut balls for labor pain, delivery duration, and mode of delivery: a meta-analysis of randomized controlled trials
Source: PeerJ. 2026 Apr 2;14:e21062. doi: 10.7717/peerj.21062 (PMC13050517; doi:10.7717/peerj.21062)
Supplement: Supplemental Information 4 [file peerj-14-21062-s004.pdf]

List of excluded studies with reasons for exclusion.

First author	Year of publication	Title
**1. Not RCT (n = 5)**		
Annamalai[1]	2024	Effectiveness of Swiss Ball Exercise Compared to Mat Exercise on Pelvic Girdle Pain in Antenatal Women
Hickey[2]	2019	Effect of Peanut Ball and Position Changes in Women Laboring With an Epidural
Jha[3]	2023	The Effect of Birthing Ball Exercises on Labor Pain and Labor Outcome Among Primigraviade Parturient Mothers at a Tertiary Care Hospital
Sundaram[4]	2022	Birthing ball technique and Sacral Massage on Maternal and fetal wellbeing: An Experimental-Pilot report
Taavoni[5]	2016	Birth ball or heat therapy? A randomized controlled trial to compare the effectiveness of birth ball usage with sacrum-perineal heat therapy in labor pain management
**2. Not English (n = 4)**		
Tian[6]	2013	Effects of Birth Ball Exercises on Labor Pain and Childbirth Satisfaction
Delgado-García[7]	2012	Randomised controlled clinical trial to determine the effects of the use of birth balls during labour
Fernández-Arranz[8]	2019	Birthing ball versus pethidine and haloperidol in satisfaction with childbirth
Lopes TC[9]	2003	The use of the birth ball in promoting the upright position during labor in first deliveries
**3. Ineligible intervention (n = 2)**		
ÇEtİNkaya[10]	2024	Effect of Chewing Gum and Stress Ball on Labor Pain, Duration of Labor, and Birth Satisfaction: a Randomized Controlled Study
Wu[11]	2022	Effect of the labour roadmap on anxiety, labour pain, sense of control, and gestational outcomes in primiparas
**3. Irrelevant outcome (n = 2)**		
Kamath[12]	2024	Effectiveness of peanut ball intervention on childbirth experiences, maternal satisfaction and behavioural responses among pregnant mothers: a randomised control trial
Sharifipour[13]	2022	The effect of delivery ball and warm shower on the childbirth experience of nulliparous women: a randomized controlled clinical trial

**4. Conference abstract (n = 1)**		
Evans[14]	2016	Use of Peanut Labor Ball for Pelvic Positioning for Nulliparous Women Following Epidural Anesthesia
**5. Thesis (n = 1)**		
BE[15]	2015	Estudio experimental controlado y aleatorizado sobre la efectividad y seguridad del uso de la pelota de parto durante el trabajo de parto
**5. Irrelevant topic (n = 1)**		
Supriatiningsih[16]	2019	Effect of pelvic rocking exercise using the birth ball on fetal lie, attitude, and presentation

References

1. Annamalai S, Jayakumar S, Nirmala J G. Effectiveness of Swiss Ball Exercise Compared to Mat Exercise on Pelvic Girdle Pain in Antenatal Women. ***Indian journal of physiotherapy & occupational therapy***. 2024;18:333‐339.
2. Hickey L, Savage J. Effect of Peanut Ball and Position Changes in Women Laboring With an Epidural. ***Nurs Womens Health***. 2019;23(3):245-252.
3. Jha S, Vyas H, Nebhinani M, Singh P, T D. The Effect of Birthing Ball Exercises on Labor Pain and Labor Outcome Among Primigraviade Parturient Mothers at a Tertiary Care Hospital. ***Cureus***. 2023.
4. Sundaram M, Bhuvaneswari G, Anand C. Birthing ball technique and Sacral Massage on Maternal and fetal wellbeing: An Experimental-Pilot report. ***Cardiometry***. 2022(23):173
EP - 178.
5. Taavoni S, Sheikhan F, Abdolahian S, Ghavi F. Birth ball or heat therapy? A randomized controlled trial to compare the effectiveness of birth ball usage with sacrum-perineal heat therapy in labor pain management. ***Complementary therapies in clinical practice***. 2016;24:99
EP - 102.
6. 田淑惠，高千惠，林宽佳，张静宜，高美玲. 生产球运动计画对孕产期妇女生产疼痛及生产满意度之成效. ***护理暨健康照护研究***. 2013;9(1):13-22.
7. Delgado-García B E, Orts-Cortés M I, Poveda-Bernabeu A, Caballero-Pérez P. [Randomised controlled clinical trial to determine the effects of the use of birth balls during labour]. ***Enfermeria Clinica***. 2012;22(1):35-40.
8. Fernández-Arranz J, Pedraz-Marcos A, Palmar-Santos A M, Moro-Tejedor M N. Birthing ball versus pethidine and haloperidol in satisfaction with childbirth. ***Enfermeria Clinica (English Edition)***. 2019;29(4):234-238.
9. Lopes TC, Madeira LM, S C. The use of the birth ball in promoting the upright position during labor in first deliveries. ***Rev Min Enfrevminenf***. 2003;7(2):134-139.
10. ÇEtİNkaya Ş, DurmuŞ A. Effect of Chewing Gum and Stress Ball on Labor Pain, Duration of Labor, and Birth Satisfaction: a Randomized Controlled Study. ***Forbes journal of medicine***. 2024;5(1):51‐58.
11. Wu N, Huang R, Shan S, Li Y, Jiang H. Effect of the labour roadmap on anxiety, labour pain, sense of control, and gestational outcomes in primiparas. ***Complementary Therapies in Clinical Practice***. 2022;46:101545.

12. Kamath P, Pai M V, Nayak B S, Upadhya R, C P, Shankar R, Bhakshi R, Noronha J A. Effectiveness of peanut ball intervention on childbirth experiences, maternal satisfaction and behavioural responses among pregnant mothers: a randomised control trial. ***Clin Epidemiol Glob***. 2024;29:101712.
13. Sharifipour P, Kheirkhah M, Rajati M, Haghani H. The effect of delivery ball and warm shower on the childbirth experience of nulliparous women: a randomized controlled clinical trial. ***Trials***. 2022;23(1):391.
14. Evans S J, Cremering M M. Use of Peanut Labor Ball for Pelvic Positioning for Nulliparous Women Following Epidural Anesthesia. ***Journal of Obstetric, Gynecologic & Neonatal Nursing***. 2016;45(3):S47.
15. BE D-G ı. Estudio experimental controlado y aleatorizado sobre la efectividad y seguridad del uso de la pelota de parto durante el trabajo de parto: Universidad de Alicante; 2015.
16. Supriatiningsih, Herlina, Wulandari L A, Retno S N, Kanedi M. Effect of pelvic rocking exercise using the birth ball on fetal lie, attitude, and presentation. ***International journal of women's health and reproduction sciences***. 2019;7(4):461 - 466.
