# Supplementary material for: Birthing balls and peanut balls for labor pain, delivery duration, and mode of delivery: a meta-analysis of randomized controlled trials
Source: PeerJ. 2026 Apr 2;14:e21062. doi: 10.7717/peerj.21062 (PMC13050517; doi:10.7717/peerj.21062)
Supplement: Supplemental Information 5 [file peerj-14-21062-s005.pdf]

Leave-one-out sensitivity analyses.

A. **Leave-one-out sensitivity analyses for birthing balls**

a. Labor pain by birthing balls

Study omitted	Estimate	[95% Conf.	Interval]
Cavalcanti, 2019	-1.9287169	-2.5048828	-1.3525511
Delgado, 2024	-1.7961146	-2.4668789	-1.1253502
Dunmez, 2023	-1.6986084	-2.2759666	-1.1212502
Erkal Aksoy, 2024	-1.8920289	-2.4850943	-1.2989634
Gau, 2011	-1.8082404	-2.3885105	-1.2279704
Gallo, 2014	-1.7058239	-2.2999995	-1.1116484
Wang, 2020	-1.8591425	-2.4681253	-1.2501599
Gallo, 2018	-1.710809	-2.3047719	-1.1168462
Taavoni, 2011	-1.8131741	-2.4271479	-1.1992004
Shirazi, 2019	-1.7308707	-2.3382547	-1.1234868
Mylod, 2024	-1.9968686	-2.4670286	-1.5267085
Sönmez, 2023	-1.8097717	-2.4354577	-1.1840856
Aktas, 2021	-1.7322043	-2.3473256	-1.117083
Combined	-1.8060027	-2.377345	-1.2346603

This table displays the leave-one-out sensitivity analysis results, which is used to assess the robustness of the pooled effect size:

- The “Study omitted” column lists each individual study that was sequentially excluded from the analysis;
- The “Estimate” column represents the re-calculated pooled effect size after removing the corresponding study;
- The “[95% Conf. Interval]” column shows the 95% confidence interval of the re-calculated effect size;
- The “Combined” row presents the original pooled effect size when all studies were included.

The results demonstrate that after omitting any single study in sequence, the

re-calculated pooled effect sizes and their 95% confidence intervals differ only slightly from the original combined results (in the "Combined" row). This indicates that the pooled analysis findings of this study have good robustness.

b. Duration of the first phase of labor by birthing balls

Study omitted	Estimate	[95% Conf.	Interval]
Delgado, 2024	-.7662816	-1.1746165	-.35794672
Dunmez, 2023	-.825553	-1.2781464	-.37295967
Erkal Aksoy, 2024	-.91533303	-1.3607223	-.46994367
Gau, 2011	-.90882319	-1.3543109	-.46333548
Shen, 2021	-.87375063	-1.3571044	-.39039692
Mathew, 2012	-.72562438	-1.1188213	-.33242747
Aslanta?, 2023	-.78047454	-1.2095188	-.3514303
Taavoni, 2011	-.97001725	-1.3673801	-.57265431
Shirazi, 2019	-.94722396	-1.364078	-.53036994
Combined	-.85618034	-1.2607254	-.45163529

c. Duration of the second phase of labor by birthing balls

Study omitted	Estimate	[95% Conf.	Interval]
Delgado, 2024	-16.72541	-27.379358	-6.071465
Dunmez, 2023	-18.412527	-30.205715	-6.6193399
Gau, 2011	-18.912817	-29.264194	-8.5614405
Shen, 2021	-18.774767	-30.454899	-7.0946331
Mathew, 2012	-15.818265	-25.999218	-5.6373124
Wang, 2020	-17.060417	-28.47298	-5.6478553
Aslanta?, 2023	-11.374781	-16.717476	-6.0320854
Shirazi, 2019	-18.839142	-29.331913	-8.3463688
Combined	-17.001283	-26.541081	-7.4614859

d. Duration of the third phase of labor by birthing balls

Study omitted	Estimate	[95% Conf.	Interval]
Dunmez, 2023	-.6500001	-1.421978	.12197785
Wang, 2020	-.02000046	-1.6882763	1.6482754
Combined	-.53889096	-1.2394949	.16171303

e. Chance of vaginal delivery by birthing balls

Study omitted	Estimate	[95% Conf.	Interval]
Delgado, 2024	1.0873764	.98176998	1.2043426
Gau, 2011	1.1072016	1.0031816	1.2220075
Shen, 2021	1.1011105	.99246407	1.2216505
Mylod, 2024	1.1062562	.99590307	1.2288373
Gallo, 2014	1.0986538	.99939096	1.2077758
Arulappan, 2014	1.0939161	.98578018	1.2139139
Mathew, 2012	1.0943971	.99451745	1.2043077
Wang, 2020	1.094046	.99169159	1.2069646
Shirazi, 2019	1.1056019	1.0020858	1.2198112
Combined	1.0987057	.99994942	1.2072154

f. Incidence of cesarean delivery by birthing balls

Study omitted	Estimate	[95% Conf.	Interval]
Delgado, 2024	.50646025	.34232062	.7493034
Shen, 2021	.52537233	.36122212	.76411724
Gallo, 2014	.53122663	.38014299	.74235678
Arulappan, 2014	.45437434	.30599156	.67471153
Mathew, 2012	.53122663	.38014299	.74235678
Wang, 2020	.50078535	.34381762	.72941577
Combined	.50990889	.36535509	.71165582

B. **Leave-one-out sensitivity analyses for peanut balls**

a. Labor pain by peanut balls

Study omitted	Estimate	[95% Conf.	Interval]
Alan Dikmen, 2024	-1.0311242	-3.4711275	1.4088792
de Sena Fraga, 2024	-1.23979	-3.2879272	.80834705
Sönmez, 2023	.05915561	-.31529775	.43360895
Combined	-.74561124	-2.1239561	.63273363

b. Duration of the first phase of labor by peanut balls

Study omitted	Estimate	[95% Conf.	Interval]
de Sena Fraga, 2024	-.87548268	-1.4053746	-.34559074
Mercier, 2018	-1.0039884	-1.3657	-.64227682
Tussey, 2015	-.69438767	-.97927308	-.40950227
Çankaya, 2025	-.8399632	-1.4153025	-.26462388
Combined	-.85480834	-1.2500963	-.45952034

c. Duration of the second phase of labor by peanut balls

Study omitted	Estimate	[95% Conf.	Interval]
de Sena Fraga, 2024	-12.539906	-29.377607	4.2977953
Tussey, 2015	-5.3119555	-9.2576389	-1.3662714
Çankaya, 2025	-15.633345	-28.568943	-2.6977465
Combined	-10.902992	-21.109547	-.69643641

d. Chance of vaginal delivery by peanut balls

Study omitted	Estimate	[95% Conf.	Interval]
Mercier, 2018	1.0940945	.90458131	1.3233116
Tussey, 2015	1.0525613	.82672548	1.3400884
de Sena Fraga, 2024	1.0826222	.87812346	1.3347448
Combined	1.079653	.90900653	1.2823347

e. Incidence of cesarean delivery by peanut balls

Study omitted	Estimate	[95% Conf.	Interval]
de Sena Fraga, 2024	.72587657	.45584795	1.1558608
Mercier, 2018	.47714725	.25438565	.89497781
Tussey, 2015	.72351819	.40808696	1.2827624
Combined	.64507313	.41461046	1.0036393
