# Supplementary material for: Birthing balls and peanut balls for labor pain, delivery duration, and mode of delivery: a meta-analysis of randomized controlled trials
Source: PeerJ. 2026 Apr 2;14:e21062. doi: 10.7717/peerj.21062 (PMC13050517; doi:10.7717/peerj.21062)
Supplement: Supplemental Information 6 [file peerj-14-21062-s006.pdf]

Subgroup analyses of birthing balls based on timing of intervention.

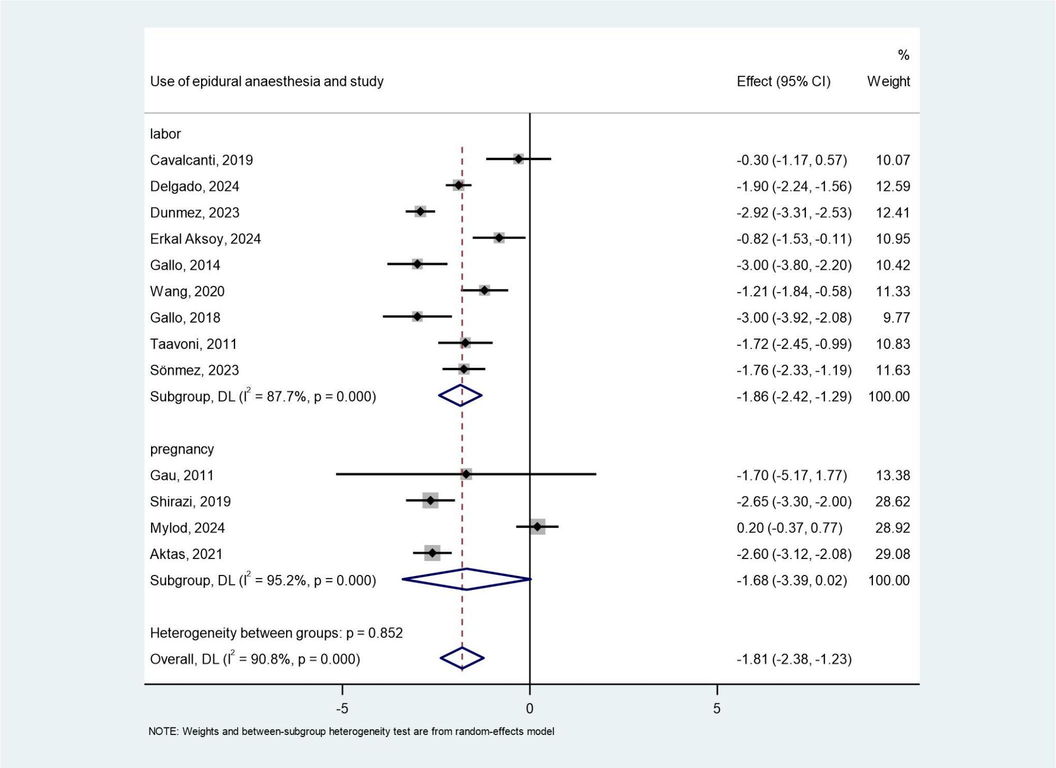

Subgroup analysis based on time of intervention for labor pain. Effects are expressed as mean difference (MD) and 95% CI.

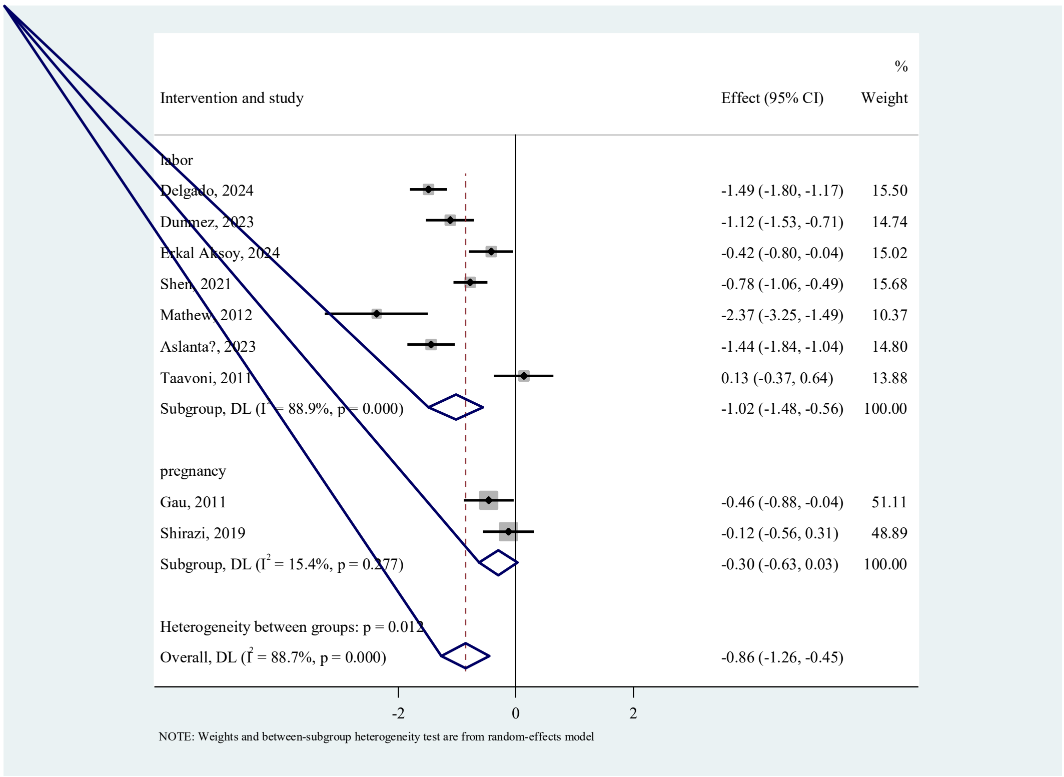

Subgroup analysis based on time of intervention for duration of the first phase of labor.

Effects are expressed as standardized mean difference (SMD) and 95% CI.

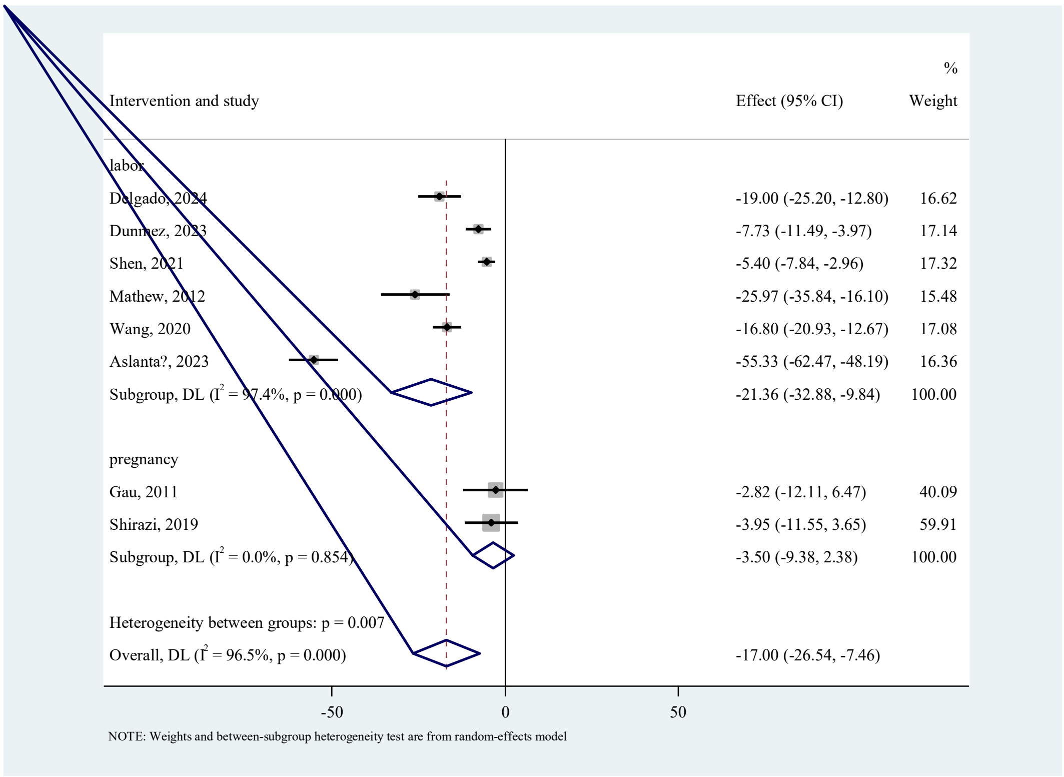

Subgroup analysis based on time of intervention for duration of the second phase of labor. Effects are expressed as mean difference (MD) and 95% CI.

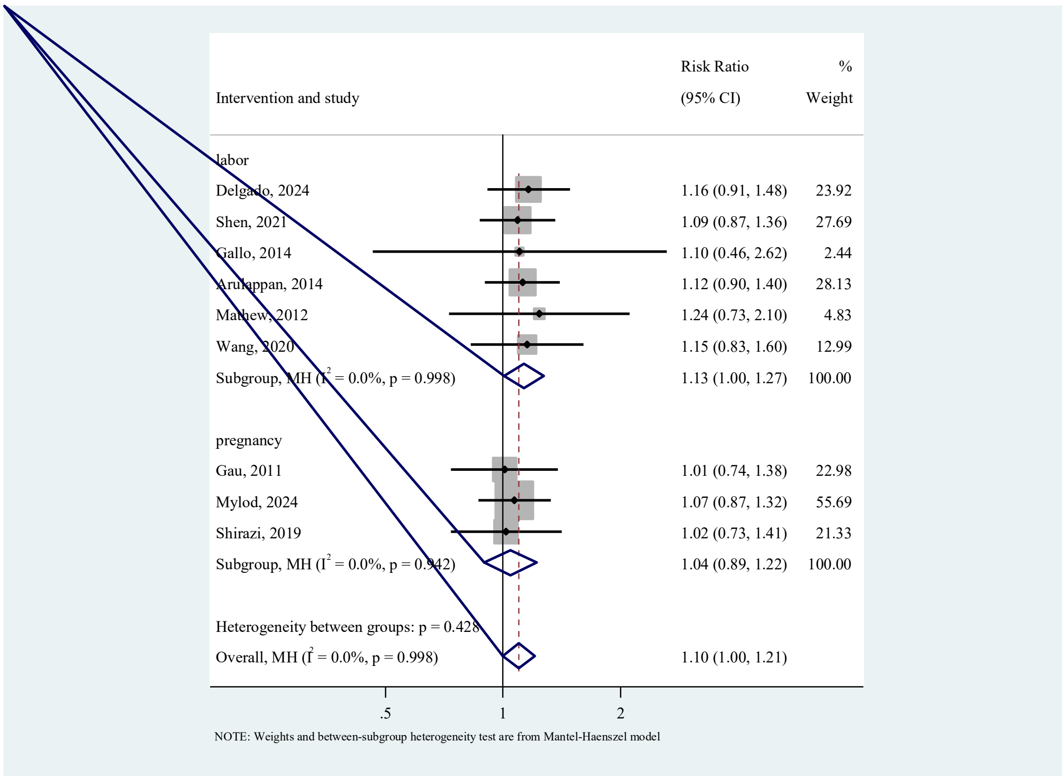

Subgroup analysis based on time of intervention for vaginal delivery. Effects are expressed as risk ratio (RR) and 95% CI.

Subgroup analyses of peanut balls based on use of analgesic.

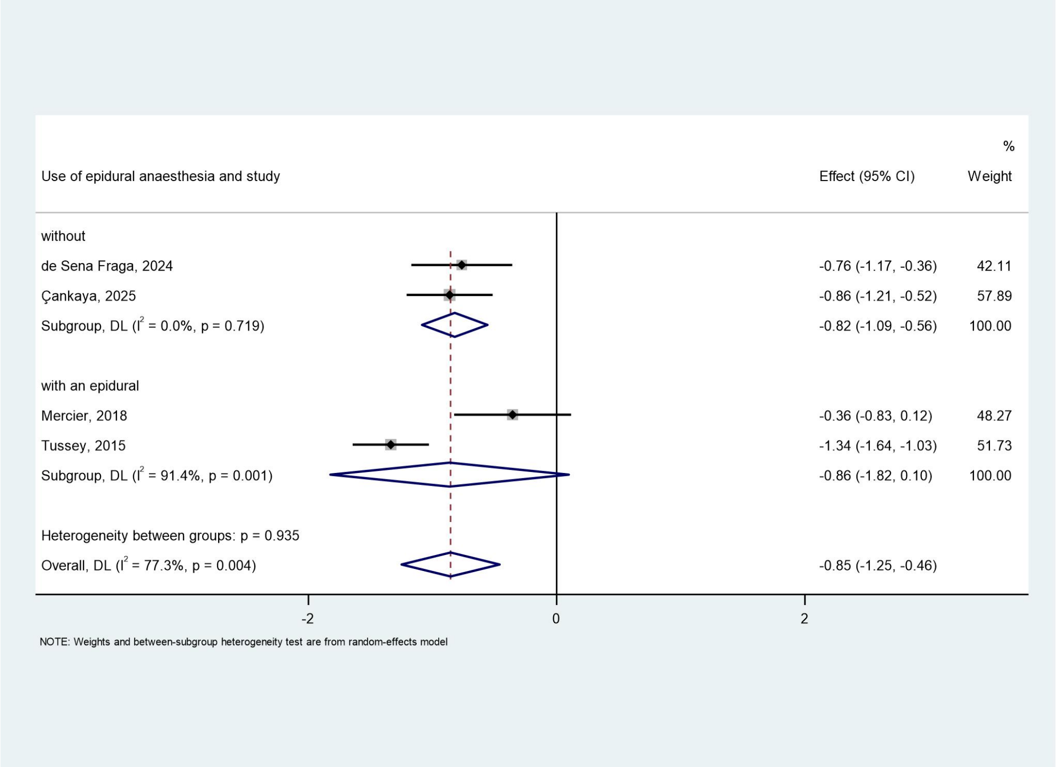

Subgroup analysis based on use of analgesic for duration of the first phase of labor. Effects are expressed as standardized mean difference (SMD) and 95% CI.
