# Supplementary material for: Birthing balls and peanut balls for labor pain, delivery duration, and mode of delivery: a meta-analysis of randomized controlled trials
Source: PeerJ. 2026 Apr 2;14:e21062. doi: 10.7717/peerj.21062 (PMC13050517; doi:10.7717/peerj.21062)
Supplement: Supplemental Information 7 [file peerj-14-21062-s007.pdf]

Funnel plot for labor pain by birthing balls.

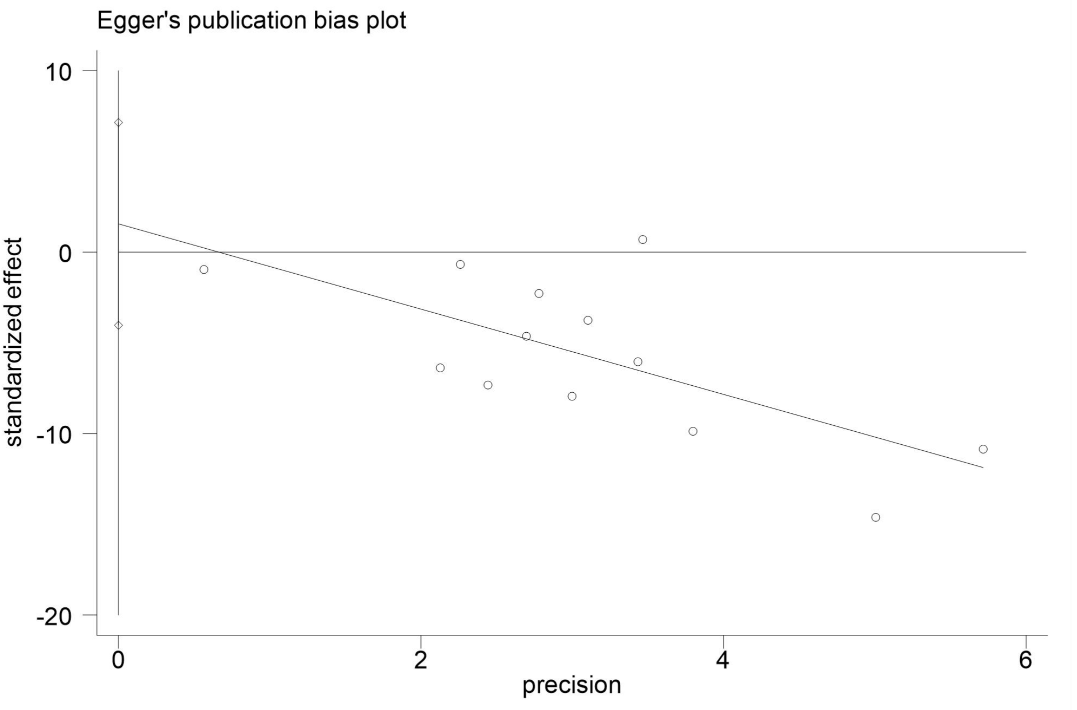

Egger's test

Std_Eff	Coef.	Std. Err.	t	P>|t|	[95% Conf.	Interval]
slope	-2.34912	.7587607	-3.10	0.010	-4.019141	-.679099
bias	1.555272	2.538543	0.61	0.553	-4.032022	7.142567
