# Supplementary material for: Birthing balls and peanut balls for labor pain, delivery duration, and mode of delivery: a meta-analysis of randomized controlled trials
Source: PeerJ. 2026 Apr 2;14:e21062. doi: 10.7717/peerj.21062 (PMC13050517; doi:10.7717/peerj.21062)
Supplement: Supplemental Information 8 [file peerj-14-21062-s008.pdf]

GRADE Profile of the Included Studies.

Certainty Assessment							No. of Patients		Certainty	Importance
No. of Studies	Study Design	Risk of Bias	Inconsistency	Indirectness	Imprecision	Other Considerations	intervention	control		
Labor pain (birthing balls)										
11	randomized trials	serious [a]	not serious	not serious	not serious	none	665	660	⊕⊕⊕◯ MODERATE	CRITICAL
Labor pain (peanut balls)										
2	randomized trials	serious [a]	not serious	not serious	serious [b]	none	155	155	⊕⊕◯◯ LOW	CRITICAL
Length of the first phase of labor (birthing balls)										
9	randomized trials	serious [a]	not serious	not serious	not serious	none	513	492	⊕⊕⊕◯ MODERATE	CRITICAL
Length of the first phase of labor (peanut balls)										
3	randomized trials	serious [a]	not serious	not serious	serious [b]	none	266	246	⊕⊕◯◯ LOW	CRITICAL
Length of the second phase of labor (birthing balls)										
8	randomized trials	serious [a]	not serious	not serious	not serious	none	482	462	⊕⊕⊕◯ MODERATE	CRITICAL

Length of the second phase of labor (peanut balls)										
2	randomized trials	serious [a]	not serious	not serious	serious [b]	none	225	206	⨁⨁◯◯ LOW	CRITICAL
Length of the third phase of labor (birthing balls)										
2	randomized trials	serious [a]	not serious	not serious	serious [b]	none	108	108	⨁⨁◯◯ LOW	CRITICAL
Spontaneous vaginal delivery (birthing balls)										
9	randomized trials	serious [a]	not serious	not serious	serious [c]	none	630	621	⨁⨁◯◯ LOW	IMPORTANT
Spontaneous vaginal delivery (peanut balls)										
3	randomized trials	serious [a]	not serious	not serious	serious [b]	none	200	186	⨁⨁◯◯ LOW	IMPORTANT
Cesarean delivery (birthing balls)										
6	randomized trials	serious [a]	not serious	not serious	not serious	none	401	400	⨁⨁⨁◯ MODERATE	IMPORTANT
Cesarean delivery (peanut balls)										

3	randomized trials	serious [a]	not serious	not serious	serious [b]	none	200	187	⊕⊕◯◯ LOW	IMPORTANT

[a] Unclear risk of bias

[b] Small sample size

[c] Confidence intervals include values favoring either treatment
