# Supplementary material for: Birthing balls and peanut balls for labor pain, delivery duration, and mode of delivery: a meta-analysis of randomized controlled trials
Source: PeerJ. 2026 Apr 2;14:e21062. doi: 10.7717/peerj.21062 (PMC13050517; doi:10.7717/peerj.21062)
Supplement: Supplemental Information 9 [file peerj-14-21062-s009.docx]

This meta-analysis on the application of birth balls in labor is primarily intended for maternity healthcare providers (including obstetricians, midwives, and labor nurses) who seek evidence-based guidance to integrate birthing and peanut balls into clinical practice. It also serves childbirth educators designing prenatal education programs, as well as pregnant individuals and their support persons interested in evidence-backed non-pharmacological pain management options during labor. Additionally, it offers valuable insights for researchers exploring further studies on maternal comfort and labor outcomes related to birthing ball use.​
